# Longitudinal social contacts among school-aged children during the COVID-19 pandemic: the Bay Area Contacts among Kids (BACK) study

**DOI:** 10.1186/s12879-022-07218-4

**Published:** 2022-03-10

**Authors:** Kristin L. Andrejko, Jennifer R. Head, Joseph A. Lewnard, Justin V. Remais

**Affiliations:** 1 Division of Epidemiology, School of Public Health, University of California, Berkeley, CA USA; 2 Division of Infectious Diseases & Vaccinology, School of Public Health, University of California, Berkeley, CA USA; 3 Center for Computational Biology, College of Engineering, University of California, Berkeley, CA USA; 4 Division of Environmental Health Sciences, University of California, Berkeley, CA USA

**Keywords:** COVID-19, SARS-COV-2, Contact survey, Contact rate, Children social networks, Reproduction number, Physical distancing, School closures

## Abstract

**Background:**

The San Francisco Bay Area was the first region in the United States to enact school closures to mitigate SARS-CoV-2 transmission. The effects of closures on contact patterns for schoolchildren and their household members remain poorly understood.

**Methods:**

We conducted serial cross-sectional surveys (May 2020, September 2020, February 2021) of Bay Area households with children to estimate age-structured daily contact rates for children and their adult household members. We examined changes in contact rates over the course of the COVID-19 pandemic, including after vaccination of household members, and compared contact patterns by household demographics using generalized estimating equations clustered by household.

**Results:**

We captured contact histories for 1,967 households on behalf of 2,674 children, comprising 15,087 non-household contacts over the three waves of data collection. Shortly after the start of shelter-in-place orders in May 2020, daily contact rates were higher among children from Hispanic families (1.52 more contacts per child per day; [95% CI: 1.14–2.04]), households whose parents were unable to work from home (1.82; [1.40–2.40]), and households with income < $150,000 (1.75; [1.33–2.33]), after adjusting for other demographic characteristics and household clustering. Between May and August 2020, non-household contacts of children increased by 145% (ages 5–12) and 172% (ages 13–17), despite few children returning to in-person instruction. Non-household contact rates among children were higher—by 1.75 [1.28–2.40] and 1.42 [0.89–2.24] contacts per child per day in 5–12 and 13–17 age groups, respectively, in households where at least one adult was vaccinated against COVID-19, compared to children’s contact rates in unvaccinated households.

**Conclusions:**

Child contact rates rebounded despite schools remaining closed, as parents obtained childcare, children engaged in contact in non-school settings, and family members were vaccinated. The waning reductions observed in non-household contact rates of schoolchildren and their family members during a prolonged school closure suggests the strategy may be ineffective for long-term SARS-CoV-2 transmission mitigation. Reductions in age-assortative contacts were not as apparent amongst children from lower income households or households where adults could not work from home. Heterogeneous reductions in contact patterns raise concerning racial, ethnic and income-based inequities associated with long-term school closures as a COVID-19 mitigation strategy.

**Supplementary Information:**

The online version contains supplementary material available at 10.1186/s12879-022-07218-4.

## Background

Physical distancing measures intending to reduce close contacts between infectious and susceptible individuals have been enacted globally to mitigate the transmission of SARS-CoV-2 [1]. The San Francisco Bay Area in California was the first region in the United States to adopt such measures, including historic, long-term transitions to remote learning for students in K-12 (ages 5–18) schools initiated as soon as March 17, 2020 with some closures lasting through the Spring 2021 semester [2]. School closures are intended to avert contacts in the school setting, eliminating within-school transmission and reducing transmission from school attendees to others in the community. However, the degree to which community transmission is reduced by school closures depends in part upon whether children interact in other settings during closures [3]. To date, the majority of social contact studies throughout the COVID-19 pandemic have been limited to adult contacts, and no studies have been conducted in the United States to quantify daily contacts among children [4–7].

The effects of stay-at-home orders and physical distancing restrictions may differ between communities. For example, while there is some evidence that school closures are associated with reductions in COVID-19 cases at the state level [8], the relative effectiveness of closures at averting contact across demographic groups is unknown [9, 10]. For example, in a sample from the United States, people in neighborhoods within the lowest income quintile reduced days at work by 6.6% following a stay-at-home order, while those in the highest income quintile reduced days at work by 13.7% [10]. Additionally, the relative effectiveness of physical distancing measures like school closures may wane over time as parents seek alternative forms of child care that involve child-child and child–adult contacts, and as children otherwise engage in activities with others outside their home [11].

Remote learning is expected to have detrimental impacts on child development, including exacerbating existing socioeconomic gaps in school achievement [11–16]. In light of these potentially severe consequences of long-term school closures, it important to understand their real-world impact on children’s contact patterns [17–19]. Here, we evaluate the evolving social contact patterns among children in the Bay Area, quantifying how social contact patterns among children varied throughout the course of the COVID-19 as well as differences across communities and demographic strata.

## Methods

### Survey methodology

**Fig. 1:**
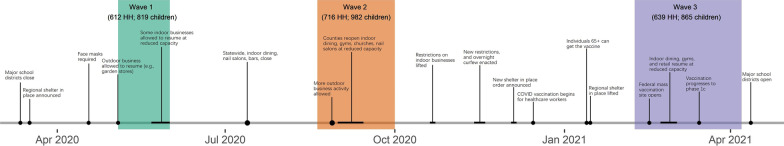
Timeline of public health restrictions in California throughout the study period

We developed a serial, cross-sectional web-based survey—Bay Area Contacts among Kids (BACK)—to capture the social contact patterns of Bay Area (California) households with children throughout the COVID-19 pandemic across three periods: May 4–June 1, 2020 (wave one), August 20–October 1, 2020 (wave two), and February 08–April 07, 2021 (wave three) (Additional file 1: File S1). Wave one represented the initial shelter-in-place period, defined as the period during which only essential business (i.e., grocery stores, pharmacies, restaurants for delivery only, hardware stores, gas stations, auto repairs, banks, laundry services, veterinary offices, public transit, and health care facilities), continued in-person and most public and private schools were closed (Fig. 1). Wave two represented an intermediate period, with permission for certain outdoor activities (swimming pools, dining, gyms, hair, and nail salons) and retail shopping, and restrictions on certain indoor activities (indoor salons, gyms, dining, worship). Public school districts remained closed, with some small, private schools open for in-person instruction. Wave three represented the period in which vaccinations had begun and restrictions further loosened, but major school districts remained closed for in-person instruction during this wave [2]. Major public-school districts in California were closed for in-person instruction for the duration of data collection.

Households in the nine Bay Area counties (Alameda, Contra Costa, Marin, Napa, San Francisco, San Mateo, Santa Clara, Solano, Sonoma) were eligible if the household contained at least one child under the age of 18. Households were recruited via a commercial survey provider (Qualtrics, Inc.) into a panel representative of the joint distribution of race/ethnicity and household income within the selected counties. One adult respondent from each sampled household responded to study questions on their own behalf, and on behalf of all children in their household. For each individual, we inquired about the number and location of non-household social contacts made within six age categories (0–4, 5–12, 13–17, 18–39, 40–64 and 65 + years) throughout the day prior to survey completion. For consistency with other studies [7], a contact was defined as an interaction within 6 feet with a non-household member lasting over 5 s. Because this study relied on adults to answer on behalf of children, and because SARS-CoV-2 is transmitted by aerosol and droplet routes of spread that do not require direct physical contact, we did not ask respondents to distinguish physical and non-physical social contacts.

We additionally collected data on demographics within each household, including race/ethnicity of members, total household income, age of each household member, and occupation status of adult respondents. Survey respondents also indicated whether members of the household experienced COVID-19-like symptoms, whether or not they felt school closures were necessary and/or useful to curb the spread of COVID-19, and their satisfaction with their ability to reduce face-to-face interactions. For each child within the household, the respondent indicated the type of schooling (remote, hybrid, in-person) their child was engaged in. For hybrid or in-person instruction, the respondent indicated physical distancing precautions implemented by the school and children’s mode of transportation to/from school. For children participating in hybrid instruction, the respondent indicated where the child went on the remote schooling days. The third wave of BACK inquired whether anyone in the household had received any doses of a COVID-19 vaccine, or if the adult respondent was planning to get vaccinated. The survey tool was translated in English and Spanish, and is included in the appendix (Additional file 1: File S1).

Our target sample size was 700 children per wave, which was the number of children needed to detect, with 80% power and 95% confidence, a difference of 0.75 mean contacts per person per day between any two racial or ethnic groups, assuming the racial and ethnic distribution of our sample matched that of the broader Bay Area population.

### Statistical analysis

To evaluate the age-specific daily frequency of non-household contacts, we generated age-structured social contact matrices by averaging the total number of contacts reported for each age group, *j*, for a given individual represented in the survey, *i*, for each wave of the BACK study. Following methods from Mossong et al. [18], the number of reported contacts was top-coded at 29. To account for potential selection bias, we created post-stratification weights reflecting the joint distributions of race/ethnicity and income of the combined population by county using the 2018 1-year American Community Survey Public Use Microdata Sample from the nine Bay Area counties. We used the joint distribution of generated 10,000 demographically weighted bootstrapped samples from each wave of data collection, clustering at the household level, to estimate age-structured social contact rates, and we present estimates representing the median, 2.5%, and 97.5% quantiles across the bootstrapped samples. We then stratified age-structured social contact matrices by each of the nine locations indicated by survey respondents whereby contact took place (home, someone else’s home, work, child-care, school, while conducting essential activities, outdoor leisure, riding or waiting for public transit, or other).

To summarize the average number of non-household contacts per person per day across each wave by household demographics we took 10,000 bootstrapped samples from BACK, clustering at the household level, and computed bootstrapped estimates of the average number of non-household contacts per person per day stratified by each of the following characteristics: age category; race/ethnicity; household size; total household income; county of residence; whether the household was a single parent household; whether there were more adults working at home due to COVID-19 physical distancing restrictions relative to the number of adults working at home before physical distancing restrictions were enacted; and (for the third wave) vaccination status.

We assessed predictors of the number of non-household contacts by fitting generalized estimating equations with robust standard errors to account for clustering at the household level. To account for overdispersion of the outcome variable (number of non-household contacts), we used a quasi-Poisson outcome distribution. Models included fixed effects for age category, race/ethnicity, combined household income, number of household members, and a changepoint term for study wave. Separate models additionally adjusted for whether adults were able to work from home during COVID-19 physical distancing restrictions and whether any adults were vaccinated in the household. To determine whether the effect of these covariates varied by study period, in three separate models, we included interactions of the study wave with age, race/ethnicity, household income, and an indicator of ability to work from home during COVID-19. Since generalized estimating equations use quasi-likelihood based inference, we were unable to assess model fit using likelihood-based methods [20]; however, our models adjusted for variables demonstrated previously to be associated with non-household contacts during non-pandemic periods [18]. Missing values for the variable indicating whether more adults worked from home during physical distancing restrictions (3.9%; 76/1967) were populated in five independent iterations of pseudo-data; pooled parameter estimates were obtained from regression models fit to the five multiply-imputed data-sets.

We examined how participant-reported contact patterns related to changes in the effective reproduction number (*R*) over time as a supplemental analysis (Additional file 1: File S2; Fig. S6).

Analyses were conducted in R (version 3.6.1) using the Amelia II package for multiple imputation, generalized estimating equations were fit using the geeM package [21, 22].

### Determining contacts among children who attended in-person class

We accounted for the number of in-person contacts students had while attending in-person school by adjusting the age-stratified contact matrices by the number of times a student switched classrooms multiplied by the average class size and an adjustment factor of 1/5 based on prior work [23], accounting for the fact that most contacts in the class setting may not be in-person contacts. We added these school-based contacts to student’s age category in their age-stratified social contact matrix. We assumed students had in-person contact with one teacher per class change, and randomly assigned a teacher aged 18–39 (30%), 40–64 (50%), or 65 + (20%) using a probability sample estimated by the proportion of public and private school teachers in approximate age categories in the United States and California [24, 25]. Lastly, we accounted for in-person contacts during travel to school by multiplying the seating capacity of an average school bus (72) by the percentage of seats full, as estimated by the parent, and the adjustment factor.

### Ethics statement

Ethics approval was obtained from the Office for Protection of Human Subjects at the University of California, Berkeley (Protocol Number: 2020-04-13180).

## Results

### Enrollment

We surveyed 1,967 total households (612 wave one, 716 wave two, 639 wave three) on behalf of 2,674 total children (819 wave one, 982 wave two, 865 wave three) (Table 1) who reported 15,087 non-household contacts. The composition of households in BACK was representative of the joint distribution of race and household income in Bay Area. The majority of primary adult respondents within a given household identified as white (56%, 1098/1967), non-Hispanic (80%, 1578/1967), multi-parent (90%, 1772/1967) with a combined household income less than $150,000 annually (64%, 1260/1967) (Table 1). The average household size was 3.7 (SD = 1.07). Most households resided in urban counties including Alameda county (27%, 5529/1967), Santa Clara county (21%, 411/1967) or San Francisco County (20% 388/1967). The average household size was slightly smaller than the average household size for the general population of Bay Area households with children (4.2) [26]. The proportion of multi-parent households in our sample was higher than that seen in the general population (60%) [27].

**Table 1:** Characteristics of study participants by household, stratified by study wave

	Total (*N* = 1967 households)	Wave 1 (*N* = 612 households)	Wave 2 (*N* = 716 households)	Wave 3 (*N* = 639 households)
Number of household members				
Mean (SD)	3.77 (1.07)	3.71 (0.979)	3.82 (1.08)	3.77 (1.13)
Median [Min, Max]	4.00 [2. 7]	4.00 [2.00, 6.00]	4.00 [2.00, 6.00]	4 [2. 7]
	N (%)	N (%)	N (%)	N (%)
County				
Alameda	529 (26.9)	218 (35.6)	167 (23.3)	144 (22.5)
Contra Costa	298 (15.1)	121 (19.8)	81 (11.3)	96 (15.0)
Marin	22 (1.1)	4 (0.7)	11 (1.5)	7 (1.1)
Napa	14 (0.7)	5 (0.8)	3 (0.4)	6 (0.9)
San Francisco	388 (19.7)	69 (11.3)	146 (20.4)	173 (27.1)
San Mateo	153 (7.8)	42 (6.9)	69 (9.6)	42 (6.6)
Santa Clara	411 (20.9)	108 (17.6)	173 (27.1)	130 (20.3)
Solano	68 (3.5)	22 (3.6)	32 (4.5)	14 (2.2)
Sonoma	84 (4.3)	23 (3.8)	34 (4.7)	27 (4.2)
Race				
White Alone	1098 (55.8)	341 (55.7)	413 (57.7)	344 (53.8)
Black or African American Alone	147 (7.5)	56 (9.2)	45 (6.3)	46 (7.2)
Asian alone	510 (25.9)	159 (26.0)	171 (23.9)	180 (28.2)
American Indian or Alaskan Native	13 (0.7)	4 (0.7)	3 (0.4)	6 (0.9)
Native Hawaiian or Pacific Islander alone	5 (0.3)	2 (0.3)	2 (0.3)	1 (0.2)
Some other race alone	103 (5.2)	23 (3.8)	44 (6.1)	36 (5.6)
Two or more races	91 (4.6)	27 (4.4)	38 (5.3)	26 (4.1)
Hispanic				
Non-Hispanic household	1578 (80.2)	496 (81.0)	582 (81.3)	500 (78.2)
Hispanic household	389 (19.8)	116 (19.0)	134 (18.7)	139 (21.8)
Household income				
Less than $19,999	110 (5.6)	34 (5.6)	40 (5.6)	36 (5.6)
$20,000 to $39,999	158 (8.0)	51 (8.3)	54 (7.5)	53 (8.3)
$40,000 to $59,999	188 (9.6)	53 (8.7)	71 (9.9)	64 (10.0)
$60,000 to $79,999	205 (10.4)	61 (10.0)	75 (10.5)	69 (10.8)
$80,000 to $99,999	205 (10.4)	58 (9.5)	72 (10.1)	75 (11.7)
$100,000 to $149,999	394 (20.0)	107 (17.5)	140 (19.6)	147 (23.0)
$150,000 or more	707 (35.9)	248 (40.5)	264 (36.9)	195 (30.5)
Multi-parent household				
Yes	1772 (90.1)	555 (90.7)	655 (91.5)	562 (87.9)
No	195 (9.9)	57 (9.3)	61 (88.5)	77 (12.1)
Weekday of reported contacts				
Weekday	1611 (81.9)	510 (83.3)	615 (85.9)	486 (76.1)
Weekend	356 (18.1)	102 (16.7)	101 (14.1)	153 (23.9)
Work from home^1^				
Adults work from home	1039 (54.9)	437 (74.2)	329 (48.0)	273 (44.3)
Adults do not work from home	852 (45.1)	152 (25.8)	357 (52.1)	343 (55.7)

Wave 1 corresponds to data collected between May 4–June 1 2020; Wave 2 was collected August 20–October 1 2020; Wave 3 was collected February 8– April 7, 2021

^1^Due to occasional missing values in this variable across each wave of data collection, the numbers do not sum to the total number of surveyed households across each wave. Percentages are calculated out of all households who completed this question in each survey

### Participant perceptions

Sixteen percent (311/1966) of respondents reported that at least one person in their household experienced COVID-19 symptoms in the two weeks prior to survey completion. Across the three waves, most households (90%, 1752/1959) stated that they agreed school closures helped reduce the number of COVID-19 cases in the community, though the proportion of households disagreeing about the utility of school closures increased from 8% (49/610) during the first wave to 12% (79/640) in the third wave. A majority (79%, 116/147) of Black households viewed school closures as helpful to reduce COVID-19 cases, as did 92% (467/509), 90% (986/1092) and 88% (342/388) of Asian, white, and Hispanic households, respectively. Of the households with combined income over $150,000, 92% (646/703) felt school closures were useful, in comparison to 88% (1106/150) of households whose combined income was less than $150,000. Of households sampled in the second and third wave, the majority (73%, 994/1349) indicated that they had greatly reduced their face-to-face interactions with others relative to the pre-pandemic era (before Shelter-In-Place orders in March 2020) and 83% (1121/1350) stated that they were satisfied with their ability to control the amount of face-to-face interaction with non-household members.

During the third wave of data collection (February 08–April 07, 2021), 40% (259/639) of households indicated that at least one member of their household had received one or more doses of a COVID-19 vaccine. Among households where no individuals have been vaccinated, 28% (108/379) indicated they were unsure about receiving or unwilling to receive a COVID-19 vaccine. Vaccine hesitancy was higher among respondents who identified as primarily Black and households whose combined income was under $40,000 (Additional file 1: Table S1).

### Contact patterns

The mean number of non-household contacts across all age groups increased from 2.28 per person per day (95% CI: 1.99, 2.58) during the first study wave to 3.24 per person per day (95%CI: 2.93, 3.58) and 3.31 per person per day (95% CI: 3.01, 3.62) during the second and third waves, respectively (Table2; Additional file 1: Fig. S2).

**Table 2:** Mean number of contacts stratified by study wave

		Wave 1 (May 2020)		Wave 2 (September 2020)		Wave 3 (February 2021)	
		*N *= 1417 827 children, 612 households		*N *= 1689 982 children, 716 households		*N* = 1446 865 children, 639 households	
		Mean (95% CI)	Observations	Mean (95% CI)	Observations	Mean (95% CI)	Observations
All contacts		2.28 (1.99, 2.58)	1417	3.24 (2.93, 3.58)	1689	3.31 (3.02, 3.62)	1446
Age							
0-4		1.83 (1.26, 2.48)	135	2.39 (1.79, 3.1)	161	2.29 (1.82, 2.85)	183
5–12		1.03 (0.77, 1.32)	445	2.53 (2.13, 2.98)	498	2.73 (2.31, 3.22)	432
13–17		0.77 (0.53, 1.09)	236	2.1 (1.7, 2.59)	287	2.63 (2.04, 3.33)	215
18–39		4.27 (3.43, 5.18)	265	4.49 (3.82, 5.21)	336	4.22 (3.62, 4.88)	352
40–64		3.59 (2.9, 4.35)	328	4.23 (3.67, 4.83)	403	4.25 (3.59, 5.01)	262
65+		2.9 (0.4, 6)	8	1.5 (0, 6)	4	4.5 (3, 6)	2
Race							
White alone		2.25 (1.88, 2.65)	799	3.47 (3.07, 3.91)	986	3.64 (3.22, 4.09)	778
Asian alone		2.05 (1.51, 2.66)	366	2.36 (1.96, 2.83)	391	2.55 (2.11, 3.02)	405
Black or African American alone		2.28 (1.54, 3.14)	116	3.86 (2.48, 5.62)	103	4.1 (2.85, 5.57)	110
Two or More Races		3.8 (1.83, 6.2)	69	4.92 (2.73, 7.8)	91	3.17 (1.74, 5.39)	58
Some other race alone		1.85 (0.98, 2.98)	56	2.09 (1.27, 3.08)	104	3.09 (1.96, 4.49)	80
American Indian or Alaska Native alone		2.89 (0, 21)	8	4.17 (1, 10)	6	2.29 (1, 3.67)	13
Native Hawaiian or Pacific Islander alone		4 (1, 10)	3	2.12 (1, 3.25)	8	1 (1,1)	2
Hispanic							
Hispanic household		3.33 (2.49, 4.29)	261	3.55 (2.73, 4.56)	321	3.66 (3, 4.42)	312
Non-Hispanic household		2.04 (1.74, 2.34)	1156	3.16 (2.83, 3.52)	1368	3.21 (2.89, 3.56)	1134
Household size							
2		2.84 (1.8, 4.08)	98	4.05 (2.54, 5.88)	123	5.14 (3.83, 6.54)	153
3		3 (2.44, 3.63)	410	2.46 (2.06, 2.93)	443	2.89 (2.39, 3.46)	376
4		1.81 (1.43, 2.24)	618	3.38 (2.94, 3.86)	713	2.91 (2.49, 3.36)	572
5 or more		2.04 (1.43, 2.75)	291	3.51 (2.69, 4.55)	410	3.47 (2.89, 4.11)	345
Household income							
Less than $19,999		4.23 (2.76, 6.05)	63	3.3 (2.02, 5.14)	84	3.68 (2.5, 4.98)	85
$20,000 to $39,999		2.65 (1.66, 3.92)	106	3.82 (2.63, 5.3)	123	3.86 (2.68, 5.34)	122
$40,000 to $59,999		2.6 (1.54, 3.95)	110	3.35 (2.41, 4.51)	164	3.04 (2.21, 4.06)	140
$60,000 to $79,999		3.23 (2.12, 4.59)	132	2.52 (1.86, 3.33)	173	3.25 (2.29, 4.44)	160
$80,000 to $99,999		2.66 (1.63, 3.85)	133	3.6 (2.35, 5.3)	172	2.76 (2.08, 3.64)	172
$100,000 to $149,999		2.59 (1.87, 3.39)	265	3.43 (2.78, 4.19)	342	3.58 (2.98, 4.24)	328
$150,000 or more		1.5 (1.2, 1.85)	608	3.03 (2.58, 3.54)	631	3.18 (2.7, 3.69)	439
County							
San Francisco		4.81 (3.57, 6.23)	143	4.05 (3.2, 5.02)	337	3.45 (2.87, 4.08)	379
Marin		3.62 (0, 9.67)	9	2.65 (0.72, 6.05)	24	2.69 (0.7, 6.5)	15
Solano		2.89 (1.24, 5.08)	50	3.5 (2.3, 4.79)	76	2.72 (1.7, 3.71)	28
Sonoma		2.84 (1.27, 5)	51	3.08 (2.11, 4.13)	93	3.19 (2, 4.52)	72
San Mateo		2.81 (1.67, 4.21)	98	2.42 (1.78, 3.15)	162	2.36 (1.66, 3.15)	105
Napa		2.1 (0.57, 5.33)	14	1.5 (1, 2)	6	4.85 (1.22, 10.5)	16
Santa Clara		1.88 (1.3, 2.56)	233	3 (2.48, 3.61)	397	3.21 (2.61, 3.89)	288
Alameda		1.8 (1.43, 2.23)	516	2.99 (2.39, 3.67)	396	3.52 (2.85, 4.29)	325
Contra Costa		1.74 (1.23, 2.36)	303	3.47 (2.41, 4.94)	198	3.33 (2.62, 4.1)	218
Adults working from home^1^							
Same or less		3.67 (2.92, 4.48)	325	3.38 (2.94, 3.87)	819	3.51 (3.09, 3.96)	767
More adults working from home		1.79 (1.51, 2.09)	1037	2.96 (2.54, 3.45)	803	3.02 (2.59, 3.49)	627
Vaccination status^1^							
0 household members vaccinated		–	–	–	–	2.64 (2.32, 2.97)	868
≥1 household member vaccinated		–	–	–	–	4.32 (3.78, 4.89)	577

We took 10,000 bootstrapped estimates from each wave of BACK, clustering at the household level, and computed bootstrapped mean estimates within each strata. We summarized the point estimate and 95% CI as the 2.5 and 97.5% quantiles of the distribution of bootstrapped estimates

^1^Does not sum to the number of individuals since this was not a required question

The average daily contact rate increased across the three study waves, with the lowest rates of contact among all age groups observed during the strictest period of physical distancing measures (wave one). In the first wave, non-household contacts were driven by younger working aged adults (18–39 years) who had on average 4.27 contacts per person per day. In comparison, school aged children including teenagers aged 13–17 years and young children aged 5–12 year reported an average of 0.77 and 1.03 contacts per child per day during the first wave, respectively (Table2).

Children aged 5–12 increased contacts across waves, from an average of 1.03 (95% CI: 0.77, 1.32) contacts per person per day in wave one to 2.53 (95% CI: 2.13, 2.98) and 2.73 (95% CI: 2.31, 3.22) contacts per person per day in waves two and three, respectively (Table2). Likewise, children aged 13–17 increased from an average of 0.77 (95% CI: 0.53, 1.09) contacts per child per day in wave one to 2.10 (95% CI: 1.7, 2.59) and 2.63 (95% CI: 2.04, 3.33) contacts per child per day in waves two and three, respectively. Despite these increases, the majority of parents reported that their child did not attend in-person instruction throughout all waves of data collection (Additional file 1: Fig. S1; Table S2; Fig. S3). Only 8% (80/977) and 9% (79/852) of children in the second and third wave, respectively, were reported to have attended in-person instruction. This suggests that increases in non-household contacts among children reflected contacts occurring outside of school settings. Indeed, among school-aged children, contacts increased across all locations examined except essential activities, but were especially frequent at their own home, someone else’s home, and during outdoor leisure (Additional file 1: Figs. S1 and S3). Contact patterns among both school-aged children and adults remained similar between the second and third wave of data collection.

**Fig. 2:**
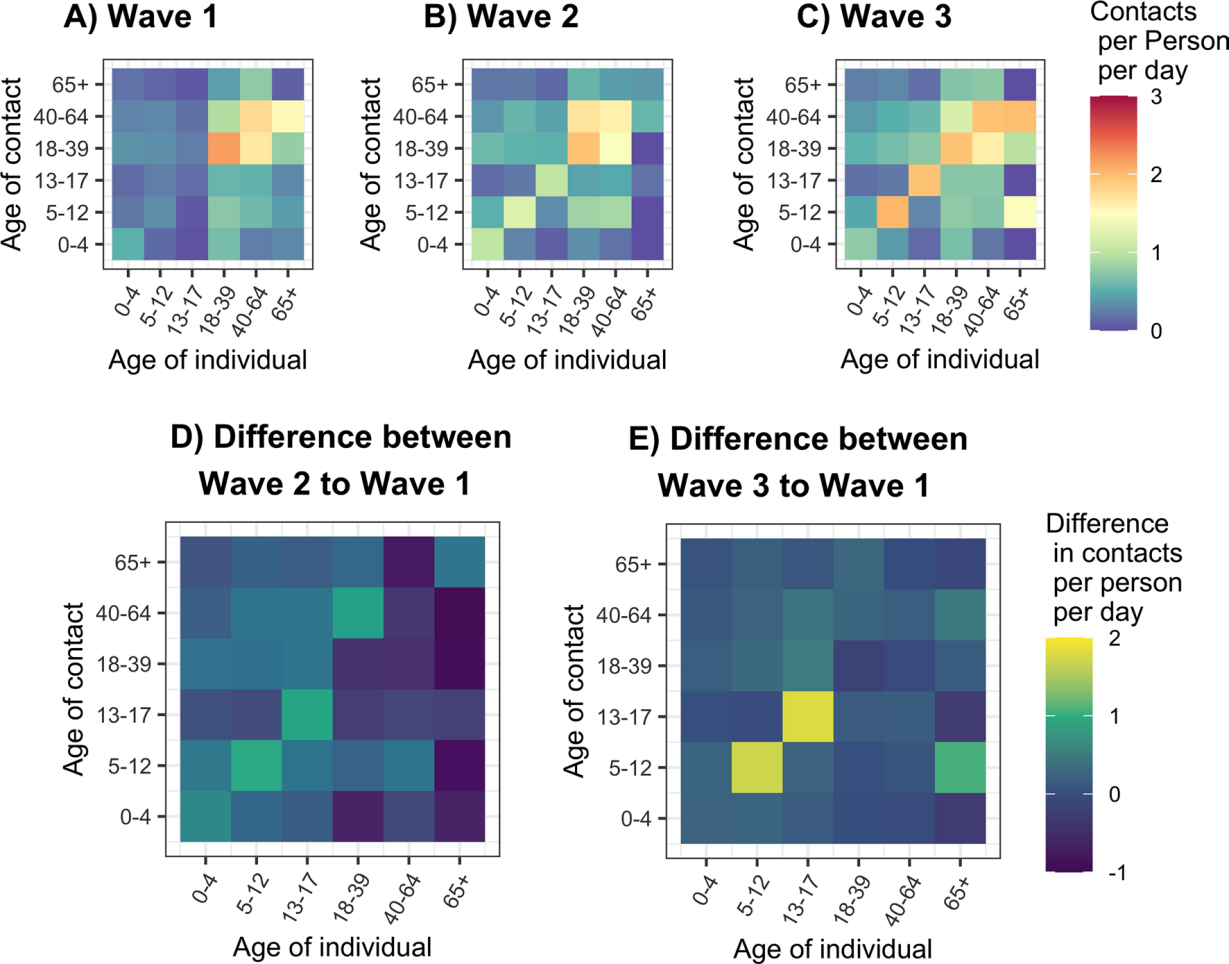
Age-structured social contact rates during the first, second and third wave of data collection. Contact matrices were generated by taking 10,000 bootstrapped samples from each wave of data collection, clustering at the household level. D-E represent the absolute difference in the number of contacts between BACK study periods

Age-structured contact matrices also revealed differences in the age-mixing behavior of respondents (Fig. 2; Additional file 1: Fig. S5). During the first wave, when all Bay Area K-12 public schools were closed for in-person instruction, child-to-child interaction was minimized, with children 5–12 equally as likely to interact with another child their age as an adult aged 18–39 years, and children 13–17 years were most likely to interact with an adult aged 18–39 years. Adults appeared to mix mainly with other adults either at work or while performing essential activities like grocery shopping (Additional file 1: Figs.S1 and S3). During the second and third waves, participants mixed with individuals of similar age at a greater frequency relative to the first wave, with these interactions primarily reported to occur at work, home, or school. Observed increases in non-household contacts between children in the second and third wave of BACK mainly occurred at home. Contacts between children and older adults did not appear to increase during periods of strict social distancing, but were higher amongst children whose parents were unable to work from home during the first wave (Additional file 1: Fig. S4).

### Determinants of non-household contacts

Across all study waves, rates of contact with non-household members varied in association with household income, ability of adults to work from home during COVID-19 physical distancing restrictions, household size, parental vaccination status, and county of residence. We estimated that individuals from households where adults were able to work from home during the period experienced 19% (95% CI: 7, 30%) fewer non-household contacts compared to households where adults were unable to work from home (Table 3). Similarly, we estimated that individuals from households whose combined income was over $150,000 had 18% (95% CI: 6, 28%) fewer non-household contacts compared to those in households with a combined household income less than $150,000. Individuals from single-parent households had 48% (95% CI: 16, 89%) more non-household contacts compared to those from multi-parent households.

**Table 3:** We fit generalized estimating equations with robust standard errors and quasi-poisson outcome distribution to estimate predictors of the total number of non-household contacts amongst all households surveyed in the study (N = 1967)

Participant characteristics	Count Ratio (95% CI)			
	Unadjusted	Adjusted		
		Model 1	Model 2	Model 3
Age				
0–4	0.51 (0.44,0.6)	0.52 (0.44,0.60)	0.52 (0.44,0.61)	0.52 (0.45,0.6)
5–12	0.48 (0.42,0.54)	0.49 (0.43,0.55)	0.49 (0.43,0.55)	0.49 (0.43,0.55)
13–17	0.46 (0.39,0.54)	0.47 (0.40,0.55)	0.47 (0.41,0.55)	0.47 (0.4,0.55)
18–39	Ref	Ref	Ref	Ref
40–64	0.98 (0.87,1.11)	1.02 (0.90,1.16)	1.03 (0.9,1.17)	1.02 (0.9,1.16)
65 +	0.87 (0.52,1.48)	0.83 (0.48,1.42)	0.84 (0.48,1.43)	0.82 (0.47,1.41)
Race				
White	Ref.	Ref.	Ref.	Ref..
Asian	0.75 (0.64,0.86)	0.77 (0.67,0.90)	0.79 (0.68,0.92)	0.77 (0.67,0.89)
Black or African American	1.08 (0.86,1.36)	0.98 (0.78,1.25)	0.96 (0.76,1.22)	0.99 (0.79,1.25)
Some other race alone	0.80 (0.62,1.02)	0.65 (0.5,00.85)	0.66 (0.5,0.86)	0.66 (0.51,0.87)
Two or more races	1.28 (0.93,1.75)	1.11 (0.81,1.52)	1.13 (0.83,1.55)	1.13 (0.83,1.55)
Hispanic	1.24 (1.07,1.43)	1.17 (0.99,1.38)	1.16 (0.99,1.37)	1.14 (0.97,1.34)
Household income ≥ $150,000	0.79 (0.69,0.89)	0.82 (0.72,0.94)	0.84 (0.73,0.96)	0.81 (0.71,0.93)
Number of household members	0.98 (0.92,1.04)	1.07 (1.01,1.14)	1.08 (1.02,1.14)	1.07 (1.01,1.14)
Single Parent	1.45 (1.17,1.79)	1.48 (1.16,1.89)	1.45 (1.14,1.85)	1.47 (1.15,1.87)
Wave				
Wave 1–May 2020	Ref.	Ref.	Ref.	Ref
Wave 2–Sep 2020	1.36 (1.15,1.6)	1.25 (1.06,1.47)	1.18 (1,1.4)	1.25 (1.06,1.47)
Wave 3–Feb 2021	1.38 (1.17,1.61)	1.26 (1.08,1.48)	1.19 (1.01,1.41)	1.01 (0.84,1.21)
More adults working from home^1^	0.71 (0.63,0.8)		0.81 (0.70,0.93)	
Vaccinated household member	1.55 (1.33,1.79)			1.62 (1.35,1.94)

We fit three adjusted models, each adjusting for age, race/ethnicity, household income, number of household members, and study wave. We additionally included the predictor for whether more or less adults worked from home, and whether a household member was vaccinated in model 2 and 3, respectively

^**1**^Missing values of whether more adults work from home during shelter in place (3.9%; 76/1967%) were multiply imputed from five independent data-sets using Amelia II

**Fig. 3:**
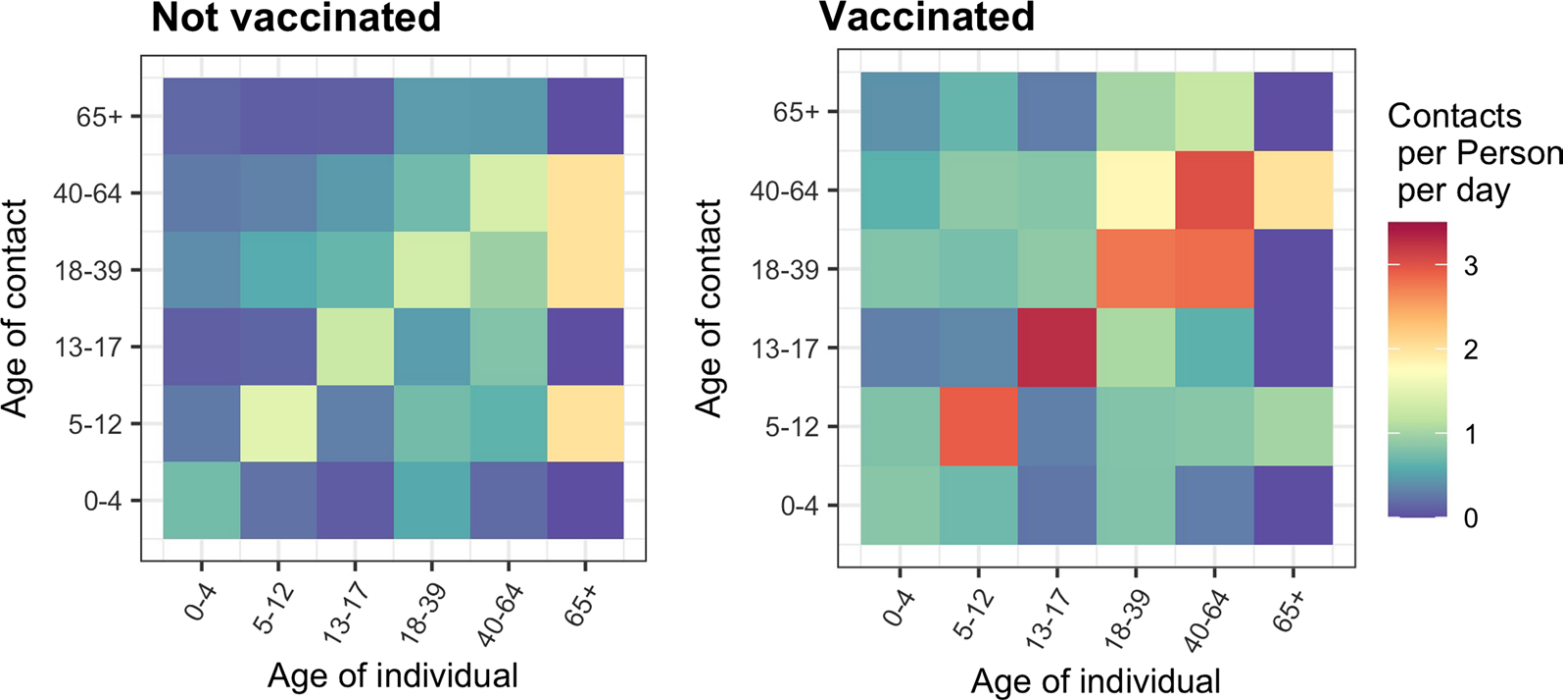
Age-structured social contact matrices stratified by household vaccination status. A vaccinated household is one in which at least one adult has been vaccinated with a COVID-19 vaccine product. No children were eligible for vaccination during the study period. Data are restricted to wave three

In the third wave of data collection, individuals from households where at least one individual was vaccinated against COVID-19 exhibited 62% (95% CI: 35, 94%) more non-household contacts compared to unvaccinated households. Age-structured contact matrices stratified by vaccination status revealed that contacts were higher both among adults and among unvaccinated children in households where at least one adult had been vaccinated (Fig. 3). In the third wave, the number of non-household daily contacts reported for a child 5–12 years and 13–17 years old from a household where one or more adults was vaccinated was on average 1.75 (95% CI: 1.28, 2.40) and 1.42 (95% CI: 0.89, 2.24) contacts per person per day higher, respectively, than their similar-aged counterparts in an unvaccinated household.

**Fig. 4:**
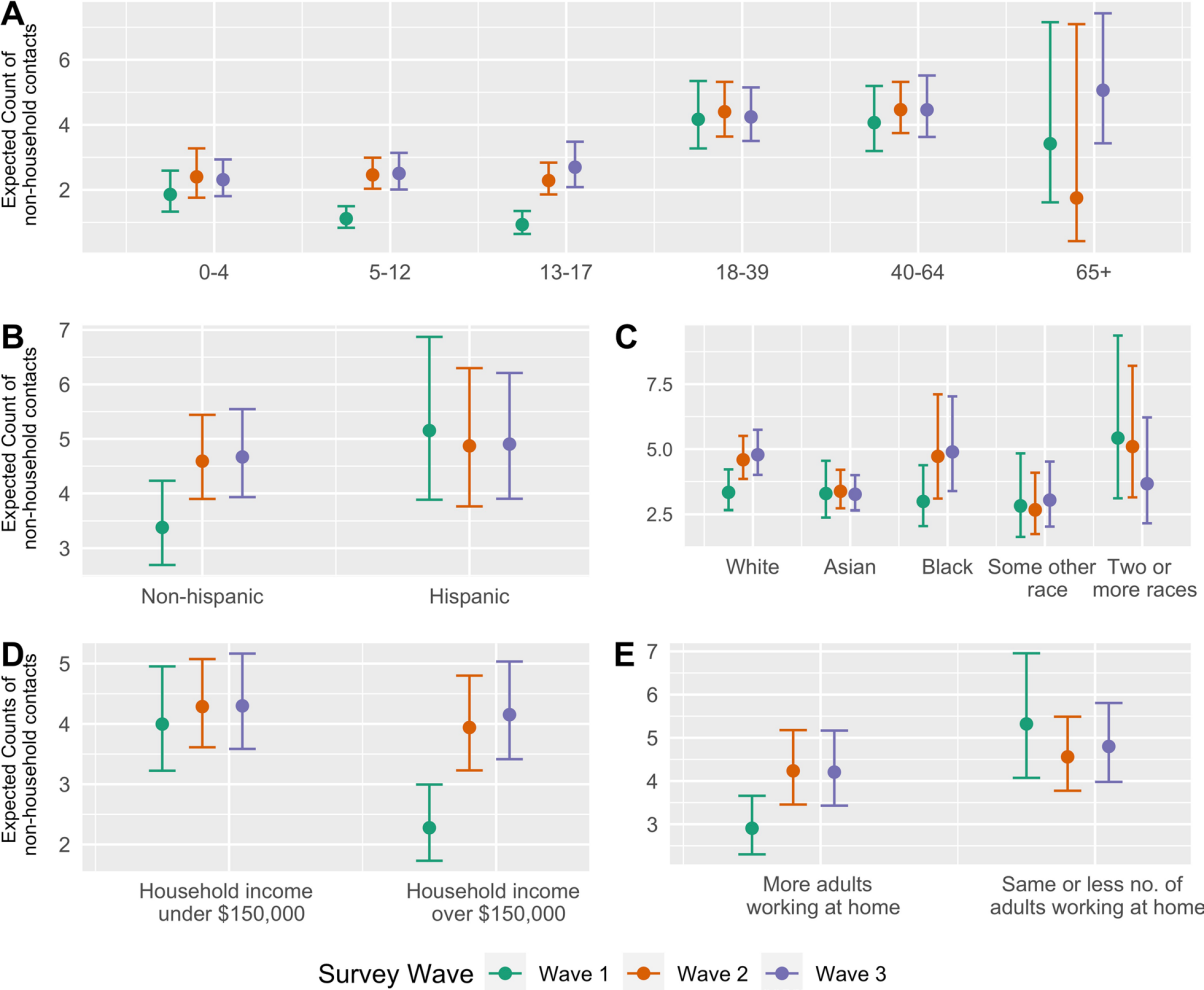
Model-based estimates of social contact by study wave and characteristics. We fit generalized estimating equations with robust standard errors to estimate how predictors of the total number of non-household contacts varied across the three BACK study waves. Plots estimate the expected count of non-household contacts with models interacting study wave with **A** age **B** whether household identifies as Hispanic **C** race/ethnicity **D** combined household income and **E** indicators of ability of adults to work from home during COVID-19 physical distancing restrictions. All models adjust for age, race/ethnicity, household count, and household income

Socioeconomic differences in rates of contact with non-household members were more pronounced in the first wave of data collection than during subsequent waves. We estimated Hispanic households had on average 1.52 (95% CI: 1.13, 2.03) more non-household contacts per person per day than non-Hispanic households in the first wave of data collection, though this difference between Hispanic and non- Hispanic households attenuated during the second and third waves (Fig. 4; Additional file 1: Table S3). Similarly, we found the estimated benefit—expressed as a reduction of non-household contacts—due to having parents working from home was most prominent during the first wave of data collection, as households where adults were unable to work from home had on average 1.82 (95% CI: 1.40, 2.40), 1.07 (95% CI: 0.82, 1.32), and 1.14 (95% CI: 0.94, 1.39) more contacts per person per day than households with adults at home during the first, second, and third wave, respectively. Across each study wave, we found that households with combined income under $150,000 had more contacts than households with combined income over $150,000, with lower income households experiencing on average 1.75 (95% CI: 1.33, 2.33), 1.08 (95% CI: 0.89, 1,33), 1.04 (95% CI: 0.84, 1.26) more contacts per person per day than higher income household during the first, second, and third wave, respectively.

## Discussion

We examined the variation in social contact patterns amongst school aged children in the California Bay Area during a period spanning strict lockdown to relaxation of some restrictions to implementation of COVID-19 vaccines. Strict physical distancing measures enforced during wave one (May 2020) were associated with the lowest age-assortive contact rates in comparison to subsequent periods of data collection, particularly among and between children at school or other childcare settings. These findings align with contact estimates elsewhere, which have found reductions in age-assortative contacts between school-aged children in China, Greece, Luxembourg, and the Netherlands during the initial April 2020 wave of the COVID-19 pandemic [6, 28–30].

After partial relaxation of physical distancing measures in the Bay Area, contact increased among school-aged children belonging to differing households. We found that teenagers aged 13–17 experienced the largest absolute increase in non-household contacts between the first and second study wave relative to all other age groups. Data from the Netherlands revealed a similar pattern: while school-aged children initially experienced striking reductions in contacts during the period of strict physical distancing in April 2020, by June 2020 contacts between children had rebounded beyond that of pre-pandemic levels [30]. Taken together, these results indicate that even in the absence of the return to widespread in-person instruction in Bay Area public schools, non-household contacts among children increased. These findings suggest that while COVID-19 related school closures reduced contacts amongst school-aged children over a short period of time, school-attributable reductions in non-household contacts may wane over the course of longer-term closures, as parents are forced to find alternative forms of childcare or socializing outside the classroom resumes. In the first wave of the survey, 74% of respondents reported that more adults were working from home compared to before the pandemic; by the second and third wave, this percentage had dropped to 48% and 41%, respectively. Thus, while the increase in contacts between the first and second wave of data collection may have been primarily driven by increased social contact among children, the increase across wave three may have been attributable to declines in the proportion of parents working from home and increased contacts after COVID-19 vaccination.

Prior to the COVID-19 pandemic, few studies provided an opportunity to address how infectious disease-related school closures would impact contact among children and adolescents. Previous contact estimates during school closures in Russia found that short-term school closures weaken children’s social networks [19]. Other studies found significant reductions in school children’s contacts over the weekend and holidays in European countries [31–34].

A study among New England school children identified that teenagers did not comply with recommendations to reduce social contacts during a short-term influenza-related school closure in 2009 [3]. In contrast, we observed low rates of social contacts particularly among teenagers during the strict initial COVID-19 lockdown period, but increases in rates of age-assortative contacts in this group followed across each subsequent study period. This suggests that COVID-19 school closures were initially taken more seriously by parents than influenza-related closures. Thus, caution is warranted in extrapolating behavioral patterns of children during short-term school closures to those anticipated during unprecedented, long-term closures.

Younger children aged 0–4 tended to have more contacts than school-aged children during the initial wave of data collection, perhaps because they were too young to be left at home alone and accompanied parents on essential activities [35]. While it has been suggested that school closures may force some children to come into contact with elderly family members for childcare [12], we did not find strong evidence of increased social contact between young children and older adults during the study period. However, during the first wave of data collection, we identified that children whose parents were unable to work from home were more likely to contact older adults than children whose parents could work from home.

We also identified disparities in the ability to effectively reduce social contacts by demographic groups, particularly Hispanic households and those making under $150,000 per year. Differences in the non-household contact rates for Hispanic and non-Hispanic families were observed in the first wave of data collection, but attenuated across subsequent periods of data collection. This effect was also observed in a social contact study of adults in urban U.S. settings throughout the COVID-19 pandemic [36]. We also identified that the ability of an adult to work from home was a significant predictor of non-household contacts among children, highlighting the need to provide safe childcare support to essential workers.

COVID-19 vaccination was associated with increased social contact outside the for both vaccinated adults and their unvaccinated children. Despite the fact that most school-aged children were not age-eligible for vaccination during the study period, presence of a vaccinated household member was associated with a significant increase in age-assortative contacts between children. It is possible that the number of non-household contacts and therefore risk of acquiring a SARS-CoV-2 infection influenced both the ability and decision to get vaccinated during the study period as the general California population did not become eligible for vaccination until April 15, 2021, after data collection concluded [37]. Likewise, it is also possible that vaccinated households expanded their social networks, per CDC guidance allowing fully vaccinated individuals to interact with low-risk, unvaccinated individuals [38]. Influenza vaccination was similarly associated with expanded social contact networks in Japan [39].

A limitation of retrospective social contact surveys is that individuals may under or over-report their children’s contacts [40, 41], and it is possible that our study was affected by self-selection bias or social desirability bias as a result of using a quota based sample from an online provider instead of a probability based sample. For instance, our sample contained a higher proportion of multi-parent households and households where parents are able to work from home than the general Bay Area population [42]. However, quota-based approaches have been widely used in contact surveys administered throughout the COVID-19 pandemic, and we obtained a representative sample based on the joint distribution of race and income [7]. Additionally, we reconstructed in-person school contacts using aggregate estimates of the age-distribution of public and private school teachers in the United States; this procedure may have misrepresented the age-structured contact patterns between children and adults in the school setting. We were unable to assess absolute reductions in contacts compared to pre-pandemic levels owing to the lack of a representative U.S.-based sample of social contact prior to COVID-19 pandemic [18]. Caution is warranted in generalizing the findings of this study, given that the demographic composition of the Bay Area and compliance with physical distancing recommendations are dissimilar to other urban settings, and our study was limited to households with children. Even still, our age-structured contact matrices revealed similar patterns to other U.S.-based contact surveys administered throughout the pandemic [36].

## Conclusions

Data from BACK provides a unique opportunity to understand how interpersonal contact patterns evolve among school-aged children in the United States throughout the COVID-19 pandemic, and may be used to parameterize models evaluating the impact of various non-pharmaceutical interventions directly impacting children like school closures. School closures in California weakened the social contact network of children, yet contact reductions were less pronounced among children in lower income households and from children whose guardians are unable to work from home. These heterogeneous reductions in contact patterns raise critical racial, ethic, and income-base inequities that warrant consideration in the event of continued in-person school closures for COVID-19 mitigation. Furthermore, the waning reductions we observed in non-household contact rates of schoolchildren and their family members during these prolonged school closures suggests that this strategy may be inadequate for long-term SARS-CoV-2 transmission mitigation.

## Supplementary Information

**Additional file 1: File S1.** Survey questionnaire. **File S2.** Supplemental Analysis: R0 estimation from contact surveys. **Table S1**. COVID-19 vaccine enthusiasm among unvaccinated households. **Table S2.** Characteristics of children attending school across Wave 2 and Wave 3. **Table S3.** Model based estimates of non-household contacts. **Figure S1.** Age-structured social contact rates during the first, second and third wave of data collection. **Figure S2. **Histogram of total contacts by study wave. **Figure S3.** Average number of non-household contacts stratified by location of contact, age category, and study wave. **Figure S4.** Age-structured contact matrices stratified by household characteristic and study wave. **Figure S5.** Age-structured contact matrices populated with bootstrapped confidence intervals. **Figure S6.** R0 estimation from contact data.

## Data Availability

The datasets supporting the conclusions of this article are available in the GitHub repository: https://github.com/kristinandrejko/BACK.

## Abbreviations

SARS-CoV-2: Severe Acute Respiratory Syndrome Coronavirus 2
BACK: Bay Area Contacts among Kids
