# Supplementary material for: Longitudinal social contacts among school-aged children during the COVID-19 pandemic: the Bay Area Contacts among Kids (BACK) study
Source: BMC Infect Dis. 2022 Mar 10;22:242. doi: 10.1186/s12879-022-07218-4 (PMC8907906; doi:10.1186/s12879-022-07218-4)
Supplement: Supplementary file 1 — Additional file 1: File S1. Survey questionnaire. File S2. Supplemental Analysis: R0 estimation from contact surveys. Table S1. COVID-19 vaccine enthusiasm among unvaccinated households. Table S2. Characteristics of children attending school across Wave 2 and Wave 3. Table S3. Model based estimates of non-household contacts. Figure S1. Age-structured social contact rates during the first, second and third wave of data collection. Figure S2. Histogram of total contacts by study wave. Figure S3. Average number of non-household contacts stratified by location of contact, age category, and study wave. Figure S4. Age-structured contact matrices stratified by household characteristic and study wave. Figure S5. Age-structured contact matrices populated with bootstrapped confidence intervals. Figure S6. R0 estimation from contact data. [file 12879_2022_7218_MOESM1_ESM.pdf]

ADDITIONAL FILE 1

Title: Longitudinal social contacts among school-aged children during the COVID-19 pandemic: the Bay Area Contacts among Kids (BACK) study

Authors: Kristin L. Andrejko[1], Jennifer R. Head, MPH[1], Joseph A. Lewnard, PhD[1,2,3] Justin V. Remais, PhD[4*]

1. Division of Epidemiology, School of Public Health, University of California, Berkeley, Berkeley, California, United States
2. Division of Infectious Diseases & Vaccinology, School of Public Health, University of California, Berkeley, Berkeley, California, United States
3. Center for Computational Biology, College of Engineering, University of California, Berkeley, Berkeley, California, United States
4. Division of Environmental Health Sciences, University of California, Berkeley, Berkeley, California, United States

*Corresponding Author: Justin V. Remais, PhD
E-mail: jvr@berkeley.edu

Table of contents

File S1	Survey questionnaire…………………………………………………………………	2
File S2	Supplemental Analysis: R0 estimation from contact surveys…………………………….	13
Table S1	COVID-19 vaccine enthusiasm among unvaccinated households………………………	15
Table S2	Characteristics of children attending school across Wave 2 and Wave 3………………	16
Table S3	Model based estimates of non-household contacts ………………………………………	17
Figure S1	Age-structured social contact rates during the first, second and third wave of data collection..	18
Figure S2	Histogram of total contacts by study wave ………………………………………………..	19
Figure S3	Average number of non-household contacts stratified by location of contact, age category, and study wave. ………………………………………………………………..	20
Figure S4	Age-structured contact matrices stratified by household characteristic and study wave……………………………..…………………………………………………	21
Figure S5	Age-structured contact matrices populated with bootstrapped confidence intervals…..	22
Figure S6	R0 Estimation from Contact Data…………………………………………………………	23

File S1. COVID-19 and Schools Survey

Section 0: Consent

DESCRIPTION: You are invited to participate in a research study to understand the effect of school closures in your community. Our team is assisting public health agencies to develop computational models that understand how school closures have affected the spread of COVID-19 in your community. These models will be useful in knowing when to re-open schools and when to close schools under future outbreaks. These models depend on knowing the contact patterns of children and their families following school closures. You will be asked to fill out a form describing the number of people and their ages that you have been within 6 feet of yesterday. We ask families with children in pre-school through 12th grade to also fill out information about their children's contacts.

PROJECT TEAM: We are a team of epidemiologists, mathematicians, and engineers at UC Berkeley School of Public Health who are assisting public health professionals in their COVID-19 planning and response efforts.

TIME COMMITMENT: Your participation will take approximately 5-10 minutes to provide information about your own contact history, and about 5 additional minutes per child to provide information about the contact history of your children.

RISKS AND BENEFITS: We foresee no risks associated with this study. The benefits which may reasonably be expected to result from this study are better epidemiological models that lead to more informed school closure policies. We cannot and do not guarantee that you will receive any benefits from this study.

PAYMENTS: This is a volunteer effort; no payments are involved. Thank you for your time!

PARTICIPANT’S RIGHTS: Your participation is voluntary and you have the right to withdraw your consent or discontinue participation at any time. The alternative is not to participate. Responses are confidential and anonymous. We do not collect personally identifying information and thus cannot identify you from your responses in the data. You have the right to refuse to answer particular questions. The results of this research study may be presented at scientific or professional meetings or published in scientific journals. Your individual privacy will be maintained in all published and written data resulting from the study.

CONTACT INFORMATION:
Questions: If you have any questions, concerns or complaints about this research, its procedures, risks and benefits, contact the UC Berkeley PI contact, Justin Remais: jvr@berkeley.edu

Independent Contact: If you are not satisfied with how this study is being conducted, or if you have any concerns, complaints, or general questions about the research or your rights as a participant, please contact the UC Berkeley Office for Protection of Human Subjects to speak to someone independent of the research team at (510)-642-7461, or by email ophs@berkeley.edu. The study was approved by UC Berkeley's Institutional Review Board with protocol ID 2020-04-13180.

Having read the information above, please select one of the two options below:

[] I CONSENT to take the survey
[] I DO NOT CONSENT to take the survey

Section 1. Screening

1. Do you have at least one child grade preK-12 in your household?
   - Yes
   - No

Section 2: Demographics

1. Choose one or more races that you identify as:
   - White
   - Black or African American
   - American Indian or Alaska Native
   - Asian
   - Native Hawaiian or Pacific Islander
   - Prefer not to say
   - Other

2. Do you identify as Hispanic, Latino, or Spanish origin?
   - Yes
   - No

3. Information about income is very important to understand. Would you please give your best guess? Please indicate the answer that includes your entire household income between January 1, 2019 and December 31, 2019 before taxes.
   - Less than $19,999

- $20,000 to $39,999
- $40,000 to $59,999
- $60,000 to $79,999
- $80,000 to $99,999
- $100,000 to $149,999
- $150,000 or more
- Prefer not to say

4. What is your zip code?
   [WRITE IN]

5. How did you hear about our survey?
   - My child's school
   - Online forum (e.g. Berkeley Parents Network, Nextdoor)
   - Social Media
   - Friend
   - Local public Health Department
   - Other

Section 3: State and County

1. In which state do you currently live?

▼ Drop down with US States

2. In which county do you currently live?

▼ Drop down with California counties, only display if State = California

3. Where do you live in [XX] County

▼ Drop down with PUMS districts in California, only display if State = California

---

Section 4: Household Composition

1. How many people (including yourself) are in your household?

INCLUDE:

- everyone who is living or staying at this address for more than 2 months
- anyone else staying at this address who does not have another place to stay, even if they are at this address for 1 month or less (ex. college student who has returned home due to university/ dorm closure)

DO NOT INCLUDE :

- anyone who is living somewhere else for more than 2 months, such as a college student living away or someone in the Armed Forces on deployment
  - 1
  - 2
  - 3
  - 4
  - 5
  - 6
  - More than 6

1. Please fill out the following information about your household:

	**Age (years)**
Household member 1 (YOU!)	[WRITE IN]
Household member 2	[WRITE IN]
Household member 3	[WRITE IN]
Household member 4	[WRITE IN]
Household member 5	[WRITE IN]

Household member 6	[WRITE IN]
Household member 7	[WRITE IN]

2. In the past two weeks, have you, or anyone in your household, experienced a fever or dry cough?
   - Yes
   - No
   - Not sure/ prefer not to say

3. **BEFORE** COVID-19 related school closures, how many adults (18 years or older) typically spent the majority of school hours (8am - 3pm) at home?

INCLUDE anyone who typically works from home, is unemployed, or retired.

[WRITE IN NUMBER]

4. **AFTER** COVID-19 related school closures, how many adults (18 years or older) typically spend the majority of school hours (8am - 3pm) at home?

[WRITE IN NUMBER]

Section 5: Adult Contact Diary

The following questions will ask about yesterday {INSERT DATE}. We know it's hard to remember exactly what happened yesterday, but please give your best guess.

1. **Where did you spend the majority of your day yesterday, [INSERT YESTERDAY'S DATE]?**
   - In my home
   - At my place of work (if your place of work is your home during shelter in place, select 'In my home')
   - At someone else's home who does not run a commercial daycare
   - At a commercial daycare location
   - At an outdoor leisure location
   - Performing essential activities, such as grocery shopping, laundering clothes, or receiving health care

2. Think about people **that you do not live with** that you were **within 6 feet of** for **more than 5 seconds** yesterday ([YESTERDAY'S DATE]). How many of these people were infants, toddlers, or pre-school aged children (0-4 years)

[WRITE IN]

2a. [IF 2 > 0] In the boxes below, write the number of infants, toddlers, or pre-school aged children (0-4 years) that you were within 6 feet of for more than 5 seconds at each location

	Number of infants, toddlers, or pre-school aged children (0-4 years)
In my home	[WRITE IN]
At my place of work	[WRITE IN]
At someone else's home	[WRITE IN]
At a childcare center that has remained open during Shelter in Place	[WRITE IN]
At an outdoor leisure location	[WRITE IN]
Performing essential activities, such as grocery shopping, laundering clothes, or receiving health care	[WRITE IN]
Riding or waiting for public transit	[WRITE IN]
My child's school	[WRITE IN]
Other	[WRITE IN]

3. Think about people **that you do not live with** that you were **within 6 feet of** for **more than 5 seconds** yesterday ([YESTERDAY'S DATE]). How many of these people were young children (5-12 years)

[WRITE IN]

3a. [IF 3 > 0] In the boxes below, write the number of young children (5-12 years) that you were within 6 feet of for more than 5 seconds at each location

	Number of young children (5-12 years)
In my home	[WRITE IN]
At my place of work	[WRITE IN]
At someone else's home	[WRITE IN]
At a childcare center that has remained open during Shelter in Place	[WRITE IN]
At an outdoor leisure location	[WRITE IN]
Performing essential activities, such as grocery shopping, laundering clothes, or receiving health care	[WRITE IN]
Riding or waiting for public transit	[WRITE IN]
My child's school	[WRITE IN]
Other	[WRITE IN]

4. Think about people **that you do not live with** that you were **within 6 feet of** for **more than 5 seconds** yesterday ([YESTERDAY'S DATE]). How many of these people were teenagers (13-17 years)

[WRITE IN]

4a. [IF 4 > 0] In the boxes below, write the number of teenagers (13-17 years) that you were within 6 feet of for more than 5 seconds at each location

	Number of teenagers (13-17 years)
In my home	[WRITE IN]
At my place of work	[WRITE IN]
At someone else's home	[WRITE IN]
At a childcare center that has remained open during Shelter in Place	[WRITE IN]
At an outdoor leisure location	[WRITE IN]
Performing essential activities, such as grocery shopping, laundering clothes, or receiving health care	[WRITE IN]
Riding or waiting for public transit	[WRITE IN]
My child's school	[WRITE IN]
Other	[WRITE IN]

5. Think about people **that you do not live with** that you were **within 6 feet of** for **more than 5 seconds** yesterday ([YESTERDAY'S DATE]). How many of these people were young adults (18-39 years)

[WRITE IN]

5a. [IF 5 > 0] In the boxes below, write the number of young adults (18-39 years) that you were within 6 feet of for more than 5 seconds at each location

	Number of young adults (18-39 years)
In my home	[WRITE IN]
At my place of work	[WRITE IN]
At someone else's home	[WRITE IN]
At a childcare center that has remained open during Shelter in Place	[WRITE IN]
At an outdoor leisure location	[WRITE IN]
Performing essential activities, such as grocery shopping, laundering clothes, or receiving health care	[WRITE IN]
Riding or waiting for public transit	[WRITE IN]
My child's school	[WRITE IN]
Other	[WRITE IN]

6. Think about people **that you do not live with** that you were **within 6 feet of** for **more than 5 seconds** yesterday ([YESTERDAY'S DATE]). How many of these people were middle aged adults (40-64 years)

[WRITE IN]

6a. [IF 6 > 0] In the boxes below, write the number of middle aged adults (40-64 years) that you were within 6 feet of for more than 5 seconds at each location

	Number of middle aged adults (40-64 years)
In my home	[WRITE IN]
At my place of work	[WRITE IN]
At someone else's home	[WRITE IN]
At a childcare center that has remained open during Shelter in Place	[WRITE IN]
At an outdoor leisure location	[WRITE IN]
Performing essential activities, such as grocery shopping, laundering clothes, or receiving health care	[WRITE IN]
Riding or waiting for public transit	[WRITE IN]
My child's school	[WRITE IN]
Other	[WRITE IN]

7. Think about people **that you do not live with** that you were **within 6 feet of** for **more than 5 seconds** yesterday ([YESTERDAY'S DATE]). How many of these people were older adults (65 +)

[WRITE IN]

7a. [IF 7 > 0] In the boxes below, write the number of older adults (65+ years) that you were within 6 feet of for more than 5 seconds at each location

	Number of older adults (65 +)
In my home	[WRITE IN]
At my place of work	[WRITE IN]
At someone else's home	[WRITE IN]
At a childcare center that has remained open during Shelter in Place	[WRITE IN]
At an outdoor leisure location	[WRITE IN]
Performing essential activities, such as grocery shopping, laundering clothes, or receiving health care	[WRITE IN]
Riding or waiting for public transit	[WRITE IN]
My child's school	[WRITE IN]
Other	[WRITE IN]

8. [IF "At my place of work" is selected for ANY of 2a - 7a]: Where do you work?
    - Office building
    - Grocery store
    - Restaurant
    - Health care facility
    - Various locations, as a delivery driver or postal employee
    - Various locations, as a law enforcement officer
    - Construction site
    - Retail store
    - Public park
    - Gas station or garage
    - Child care/daycare center
    - School or tutoring agency
    - Food processing facility
    - Warehouse or manufacturing facility
    - Other

Section 6: Children Screening Questions

1. We are hoping to get information on all members of the household, especially children in pre-school - 12th grade. Are you willing to help by answering these questions for one or more of your children?

    Only answer YES if someone else in your household has not already filled out a survey for your children.
    - Yes
    - No
    - Someone else in my household has already completed the survey for my children
    - I do not have children in prek-12th grade in my household

[only display the next series of questions about kids if they answer YES above; this series will display for the number of children that they selected above]

2. How many children will you complete the survey for?
    - 1
    - 2
    - 3

- 4
- 5

3. Do you think school closures have helped reduce the number of covid-19 cases in your community (flatten the curve)?
    - Yes
    - No

4. *[only display if Yes is selected to #3]* Do you think school closures are necessary to flatten the curve?
    - Yes
    - No

5. Has your child missed any routine pediatrician appointments during the Shelter in Place order (ex. well-child check ups, yearly physical, routine childhood immunizations), either because you were unable to or unwilling to attend?

    - Yes-I was unable to attend a visit
    - Yes- I was unwilling to attend a visit
    - No- My child has not missed any appointments
    - No- My child has not had any pediatrician visits scheduled, but if they did, I would be willing to attend
    - No- My child has not had any pediatrician visits scheduled, but if they did, I would be unwilling to attend

---

Section 7: Children Contact Diary

Please answer these questions for the [first/second/third/fourth/fifth] of your school aged children.

1. How old is your child (in years?)
[Write in]

2. What type of school does your child attend?
    - Private
    - Public
    - Charter
    - Home-school
    - Other [write in]

3. Where did your child spend the majority of your day yesterday, [INSERT YESTERDAY'S DATE]?
    - In my home
    - At my place of work (if your place of work is your home, select 'In my house')
    - At someone else's home who does not run a commercial daycare
    - At a commercial daycare location
    - At an outdoor leisure location
    - Performing essential activities, such as grocery shopping, laundering clothes, or receiving health care

4. Think about people **that you do not live with** that your child was **within 6 feet of** for **more than 5 seconds** yesterday ([YESTERDAY'S DATE]). How many of these people were infants, toddlers, or pre-school aged children (0-4 years)

    [WRITE IN]

a. **[IF 4> 0]** In the boxes below, write the number of infants, toddlers, or pre-school aged children (0-4 years) that your child was within 6 feet of for more than 5 seconds at each location

	Number of infants, toddlers, or pre-school aged children (0-4 years)
In my home	[WRITE IN]
At my place of work	[WRITE IN]
At someone else's home	[WRITE IN]
At a childcare center that has remained open during Shelter in Place	[WRITE IN]

At an outdoor leisure location	[WRITE IN]
Performing essential activities, such as grocery shopping, laundering clothes, or receiving health care	[WRITE IN]
Riding or waiting for public transit	[WRITE IN]
My child's school	[WRITE IN]
Other	[WRITE IN]

5. Think about people **that you do not live with** that your child was **within 6 feet of** for **more than 5 seconds** yesterday ([YESTERDAY'S DATE]). How many of these people were young children (5-12 years)

[WRITE IN]

b. **[IF 5> 0]** In the boxes below, write the number of young children (5-12 years) that that your child was within 6 feet of for more than 5 seconds at each location

	Number of young children (5-12 years)
In my home	[WRITE IN]
At my place of work	[WRITE IN]
At someone else's home	[WRITE IN]
At a childcare center that has remained open during Shelter in Place	[WRITE IN]
At an outdoor leisure location	[WRITE IN]
Performing essential activities, such as grocery shopping, laundering clothes, or receiving health care	[WRITE IN]
Riding or waiting for public transit	[WRITE IN]
My child's school	[WRITE IN]
Other	[WRITE IN]

6. Think about people **that you do not live with** that your child was **within 6 feet of** for **more than 5 seconds** yesterday ([YESTERDAY'S DATE]). How many of these people were teenagers (13-17 years)

[WRITE IN]

6a. [IF 6 > 0] In the boxes below, write the number of teenagers (13-17 years) that that your child was within 6 feet of for more than 5 seconds at each location

	Number of teenagers (13-17 years)
In my home	[WRITE IN]
At my place of work	[WRITE IN]
At someone else's home	[WRITE IN]
At a childcare center that has remained open during Shelter in Place	[WRITE IN]
At an outdoor leisure location	[WRITE IN]
Performing essential activities, such as grocery shopping, laundering clothes, or receiving health care	[WRITE IN]

Riding or waiting for public transit	[WRITE IN]
My child's school	[WRITE IN]
Other	[WRITE IN]

7. Think about people **that you do not live with** that your child was **within 6 feet of** for **more than 5 seconds** yesterday ([YESTERDAY'S DATE]). How many of these people were young adults (18-39 years)

[WRITE IN]

7a. **[IF 57> 0]** In the boxes below, write the number of young adults (18-39 years) that your child was within 6 feet of for more than 5 seconds at each location

	Number of young adults (18-39 years)
In my home	[WRITE IN]
At my place of work	[WRITE IN]
At someone else's home	[WRITE IN]
At a childcare center that has remained open during Shelter in Place	[WRITE IN]
At an outdoor leisure location	[WRITE IN]
Performing essential activities, such as grocery shopping, laundering clothes, or receiving health care	[WRITE IN]
Riding or waiting for public transit	[WRITE IN]
My child's school	[WRITE IN]
Other	[WRITE IN]

8. Think about people **that you do not live with** that your child was **within 6 feet of** for **more than 5 seconds** yesterday ([YESTERDAY'S DATE]). How many of these people were middle aged adults (40-64 years)

[WRITE IN]

8a. [IF 8 > 0] In the boxes below, write the number of middle aged adults (40-64 years) that your child was within 6 feet of for more than 5 seconds at each location

	Number of middle aged adults (40-64 years)
In my home	[WRITE IN]
At my place of work	[WRITE IN]
At someone else's home	[WRITE IN]
At a childcare center that has remained open during Shelter in Place	[WRITE IN]
At an outdoor leisure location	[WRITE IN]
Performing essential activities, such as grocery shopping, laundering clothes, or receiving health care	[WRITE IN]
Riding or waiting for public transit	[WRITE IN]
My child's school	[WRITE IN]

Other	[WRITE IN]

9) Think about people **that you do not live with** that your child was **within 6 feet of** for **more than 5 seconds** yesterday ([YESTERDAY'S DATE]). How many of these people were older adults (65 +)

[WRITE IN]

9a. [IF 9 > 0] In the boxes below, write the number of older adults (65+ years) that your child was within 6 feet of for more than 5 seconds at each location

	Number of older adults (65 +)
In my home	[WRITE IN]
At my place of work	[WRITE IN]
At someone else's home	[WRITE IN]
At a childcare center that has remained open during Shelter in Place	[WRITE IN]
At an outdoor leisure location	[WRITE IN]
Performing essential activities, such as grocery shopping, laundering clothes, or receiving health care	[WRITE IN]
Riding or waiting for public transit	[WRITE IN]
My child's school	[WRITE IN]
Other	[WRITE IN]

[*Repeat Q's 1-9 in Section 7 depending on how many children said they would answer for]*

Section 8: Additional Questions

1. How concerned are you personally about the spread of coronavirus/ COVID-19?

- Very concerned
- Somewhat concerned
- Not very concerned
- Not at all concerned

2. How much, if at all, have you changed your face-to-face interaction with other people as a result of coronavirus/ COVID-19?

- I have greatly reduced face-to-face interaction with others
- I have somewhat reduced face-to-face interaction with others
- I have not changed
- I have increased face-to-face interaction with others

3. Are you satisfied with your ability to control how much face-to-face interaction you have with other people?

- Yes, I am satisfied with my ability to control how much face-to-face interaction I have with other people
- No, I would like to reduce face-to-face interaction with others more than I am able to
- No, I would like to increase face-to-face interaction with others more than I am able to

4. *[Only Wave Three]* Has anyone in your family received a COVID-19 vaccine?

- Yes
- No
- Not sure

5. *[Only Wave Three]* Do you plan to receive a COVID-19 vaccine when it becomes avaliable to you?

- Yes
- No
- Not sure

Section 9: Thank you message

Thank you! Your response will help schools understand the impact of school closures on COVID-19 transmission in your community!

Do you have another family member who has not taken the survey? Please invite them to participate by sharing the link here: [custom referral link]

Do you know other families who have not taken the survey? Please invite them to participate by sharing the link here: [custom referral link]

FAQ:

What will you do with this data?
Leading epidemiologists are assisting public health agencies to develop computational models that understand how school closures have affected the spread of COVID-19 in your community. These models will be useful in knowing when to re-open schools and when to close schools under future outbreaks. These models depend on knowing the contact patterns of children and their families following school closures. We urgently need volunteers to help us understand the effect of school closures in your community.

Who is behind this project?
We are a team of epidemiologists, mathematicians, and engineers at UC Berkeley School of Public Health who are assisting federal and state officials in their COVID-19 planning and response efforts

File S2. Supplemental analysis: R_0 estimation from social contact data

Methods

To examine how participant-reported contact patterns related to changes in the effective reproduction number (R) over time, we estimated R using our survey data and compared it to estimates of R calculated from incidence rates. Briefly, the basic reproduction number, R_0, represents the number of secondary infections generated from a single infected individual in a completely susceptible population. R_0 is estimated as the dominant eigenvalue of the next generation matrix, which is directly proportional to the contact matrix [1]. Assuming that the duration of infectiousness and the probability that a single contact leads to transmission does not change over the study period, the ratio of R_0 at baseline (prior to enactment of physical distancing measures) to R at the times of our survey is equal to the ratio of the dominant eigenvalues of the contact matrix at baseline and the BACK matrix. Following previous work [2, 3], we assumed that non-household, non-school contact patterns at baseline were similar to those observed in the POLYMOD data from the UK, household contact patterns at baseline were similar to those observed in BACK, and school contact patterns at baseline could be estimated from average class sizes in the Bay Area. We constructed a contact matrix at baseline by summing the household contact matrix derived from all waves of BACK, the community contact matrix defined by POLYMOD data, and a school contact matrix that scaled average class sizes by proportion of days spent in school. Following a previous meta-analysis, we assumed that R_0 followed a normal distribution with mean 2.5 and standard deviation of 0.54 [2].

We took 10,000 bootstrapped samples from BACK, clustering at the household level, and computed bootstrapped estimates of the age-structured contact matrices. We calculated the ratio between the dominant eigenvalues for the baseline matrix and each of our bootstrapped BACK matrices. We drew 10,000 random draws of R_0 at baseline from the normal distribution described. We scaled these initial estimates of R_0 with the bootstrapped ratio of dominant eigenvalues to estimate R during each study wave. This yielded a distribution of 10,000 estimates for R. We computed the point estimate and 95% CI's for R as the 2.5^{th}, 50th, and 97.5^{th} quantiles of the distribution. We compared our estimates of R to estimates calculated from a daily tracker of COVID-19 incidence for six Bay Area counties [4].

Results

Using the ratios of the dominant eigenvalue of the POLYMOD derived matrices to the dominant eigenvalues from our bootstrapped BACK matrices, we estimated R during the first, second, and third wave of the survey to be 1.18 (95% CI: 0.68, 1.74), 1.29 (95% CI: 0.75, 1.85), and 1.39 (95% CI: 0.81, 2.01), respectively. Our estimates are comparable to estimates of R calculated from COVID-19 transmission rates (**Figure S6**), although the point-estimate of our survey-derived estimates represent a slight overestimation during the second and third wave when compared to prior estimates, potentially due to prevalent mask use and vaccine administration (throughout the third wave) lowered the probability that a single contact results in infection.

Discussion/ Limitations

Estimating R from survey data can support rapid prediction of transmission, but may be limited by certain assumptions [2]. Here, we demonstrate good alignment between our survey-derived estimates of R and estimates derived from incidence data for the first wave, and an overestimate for the second and third wave. Calculating R using survey data assumes that the probability that a single contact leads to transmission does not vary over the study period. While this is commonly assumed, increased prevalence of mask wearing over the study period may

have reduced the probability of transmission given contact, and duration of contact may also have varied [2, 3]. In the third wave, increased prevalence of vaccinated individuals would have lowered the probability a single contact leads to transmission, which may explain why the third wave overestimated *R* based on contact patterns alone. Another limitation is that the degree to which children contribute to transmission of SARS-CoV-2 remains unclear, particularly amid circulation of variants of concern. Thus, if the relative contribution of transmission for children is less than that of adults, reductions in contact patterns among children will not reduce *R* to the same degree of reductions in contact patterns among adults.

References

1. Farrington CP, Kanaan MN, Gay NJ. Estimation of the basic reproduction number for infectious diseases from age-stratified serological survey data. Journal of the Royal Statistical Society: Series C (Applied Statistics). 2001;50:251–92.
2. Jarvis CI, Van Zandvoort K, Gimma A, Prem K, Auzenbergs M, O'Reilly K, et al. Quantifying the impact of physical distance measures on the transmission of COVID-19 in the UK. BMC Medicine. 2020;18:124.
3. Feehan DM, Mahmud AS. Quantifying population contact patterns in the United States during the COVID-19 pandemic. Nat Commun. 2021;12:893.
4. Worden L. COVID-19 R estimation for California. COVID-19 R estimation for California. 2021. https://ca-covid-r.info/. Accessed 15 Apr 2021.

Table S1. COVID-19 vaccine enthusiasm among unvaccinated households. During Wave 3, 379 surveyed households indicated they had not yet had an individual in their household vaccinate.

	Row total	Not willing to get a COVID Vaccine N (%)	Willing to get a COVID Vaccine N (%)
Race			
White alone	202	55 (27)	147 (73)
Asian alone	106	23 (22)	83 (78)
Black or African American alone	29	16 (55)	13 (45)
Some other race alone	21	6 (29)	15 (71)
Two or more races	17	7 (41)	10 (59)
American Indian or Alaska Native alone	3	1 (33)	2 (67)
Native Hawaiian or Pacific Islander alone	1	0 (0)	1 (100)
Hispanic	72	18 (25)	54 (75)
Not Hispanic	307	90 (29)	217 (71)f
Household Income			
Less than $19,999	25	14 (56)	11 (44)
$20,000 to $39,999	27	17 (63)	10 (37)
$40,000 to $59,999	39	18 (46)	21 (54)
$60,000 to $79,999	38	18 (47)	20 (53)
$80,000 to $99,999	52	8 (15)	44 (85)
$100,000 to $149,999	86	16 (19)	70 (81)
$150,000 or more	112	17 (15)	95 (85)
Number of household members			
2	41	15 (37)	26 (53)
3	104	25 (24)	79 (76)
4	156	38 (24)	118 (76)
5 or more	78	30 (38)	48 (62)
County			
Alameda	89	37 (42)	52 (58)
Contra Costa	54	20 (37)	34 (63)
Marin	4	1 (25)	3 (75)
Napa	1	1 (1)	0 (0)
San Francisco	100	19 (27)	81 (81)
San Mateo	25	6 (24)	19 (76)
Santa Clara	80	15 (19)	65 (81)
Solano	8	3 (38)	5 (63)
Sonoma	18	6 (33)	12 (67)
More adults working from home due to physical distancing restrictions	160	39 (24)	121 (76)

Table S2. Characteristics of children attending school across Wave 2 and Wave 3.

		**Total** (*N*=159 children)	**Wave 2** (*N*=80 children)	**Wave 3** (*N*=79 children)
Number of household members				
	Mean (SD)	3.68 (1.03)	3.63 (1.02)	3.73 (1.05)
	Median [Min, Max]	4 [2, 7]	4 [2, 7]	4 [2. 7]
County				
	Alameda	36 (22.6)	23 (28.8)	36 (26.6)
	Contra Costa	21 (13.2)	4 (5.0)	21 (13.2)
	Marin	0 (0)	0 (0)	0 (0)
	Napa	3 (1.9)	0 (0)	3 (1.9)
	San Francisco	50 (31.4)	26 (32.5)	24 (30.4)
	San Mateo	9 (8.2)	9 (11.2)	4 (5.1)
	Santa Clara	26 (16.4)	13 (16.2)	13 (16.5)
	Solano	2 (1.3)	0 (0)	2 (2.5)
	Sonoma	8 (5.0)	5 (6.2)	3 (3.8)
Race				
	White Alone	115 (72.3)	66 (82.5)	49 (62.0)
	Black or African American Alone	12 (7.5)	4 (5.0)	8 (10.1)
	Asian alone	22 (13.8)	7 (8.8)	15 (19)
	American Indian or Alaskan Native	0 (0)	0 (0)	0 (0)
	Native Hawaiian or Pacific Islander alone	0 (0)	0 (0)	0 (0)
	Some other race alone	3 (1.9)	0 (0)	3 (3.8)
	Two or more races	7 (4.4)	3 (3.8)	4 (5.1)
Hispanic				
	Non-Hispanic household	119 (74.8)	59 (73.8)	60 (75.9)
	Hispanic household	40 (25.2)	21 (26.2)	19 (24.1)
Household income				
	Less than $19,999	4 (2.5)	1 (1.2)	3 (3.8)
	$20,000 to $39,999	12 (7.5)	7 (8.8)	5 (6.3)
	$40,000 to $59,999	15 (9.4)	7 (8.8)	8 (10.1)
	$60,000 to $79,999	12 (7.5)	6 (7.5)	6 (7.6)
	$80,000 to $99,999	18 (11.3)	8 (10.0)	10 (12.7)
	$100,000 to $149,999	35 (22.0)	16 (20.0)	19 (24.1)
	$150,000 or more	63 (39.6)	35 (43.8)	28 (35.4)
Multi-parent household				
	Yes	136 (85.5)	67 (83.8)	69 (87.3)
	No	23 (14.5)	13 (16.2)	10 (12.7)

Table S3. Model-based estimates of non-household contacts. Estimates are derived from generalized estimating equations with interactions between each primary variable and the study wave. Estimates for which the covariate is not being manipulated in an interaction with study wave reflect the estimated number of non-household contacts at the reference categories of covariates included in the model (18-39 years old, non-Hispanic, white, household income under $150,000).

		**Estimated number of non-household contacts** N (95 % Confidence Interval)		
		Wave 1	*Wave 2*	*Wave 3*
Age	0-4	1.87 (1.35, 2.59)	2.4 (1.77, 3.24)	2.32 (1.81, 2.96)
	5-12	1.12 (0.83, 1.5)	2.47 (2.04, 2.97)	2.5 (2.01, 3.13)
	13-17	0.94 (0.65, 1.36)	2.28 (1.84, 2.81)	2.71 (2.09, 3.48)
	18-39	4.19 (3.26, 5.35)	4.4 (3.64, 5.31)	4.25 (3.52, 5.14)
	40- 64	4.07 (3.18, 5.21)	4.46 (3.75, 5.3)	4.47 (3.63, 5.52)
	65+	3.46 (1.64, 7.14)	1.75 (0.42, 7.3)	5.09 (3.41, 7.47)
Race/ Ethnicity	Hispanic	5.17 (3.87, 6.85)	4.88 (3.79, 6.32)	4.78 (3.99, 5.73)
	Non-Hispanic	3.39 (2.7, 4.22)	4.61 (3.9, 5.45)	4.67 (3.91, 5.56)
	White	3.34 (2.66, 4.23)	4.59 (3.85, 5.49)	4.78 (4 ,5.7)
	Asian	3.27 (2.38, 4.5)	3.36 (2.71, 4.17)	3.25 (2.65, 3.99)
	Black	2.99 (2.04, 4.33)	4.71 (3.08, 7.21)	4.87 (3.38, 7.03)
	Some other race alone	2.84 (1.64, 4.86)	2.65 (1.7, 4.09)	3.03 (2.01, 4.54)
	Two or more races	5.42 (3.1, 9.45)	5.06 (3.14, 8.22)	3.67 (2.16, 6.15)
Household income	<$150,000	4 (3.23, 4.96)	4.29 (3.61, 5.13)	4.31 (3.58, 5.18)
	≥$150,000	2.28 (1.74, 3)	3.94 (3.23, 4.84)	4.15 (3.43, 5.05)
Adults working from home	More adults at home	2.91 (2.31, 3.68))	4.24 (3.46, 5.2)	4.21 (3.41, 5.18)
	Same or less number of adults working at home	5.34 (4.07, 6.99)	4.57 (3.8, 5.5)	4.79 (4.01, 5.78)

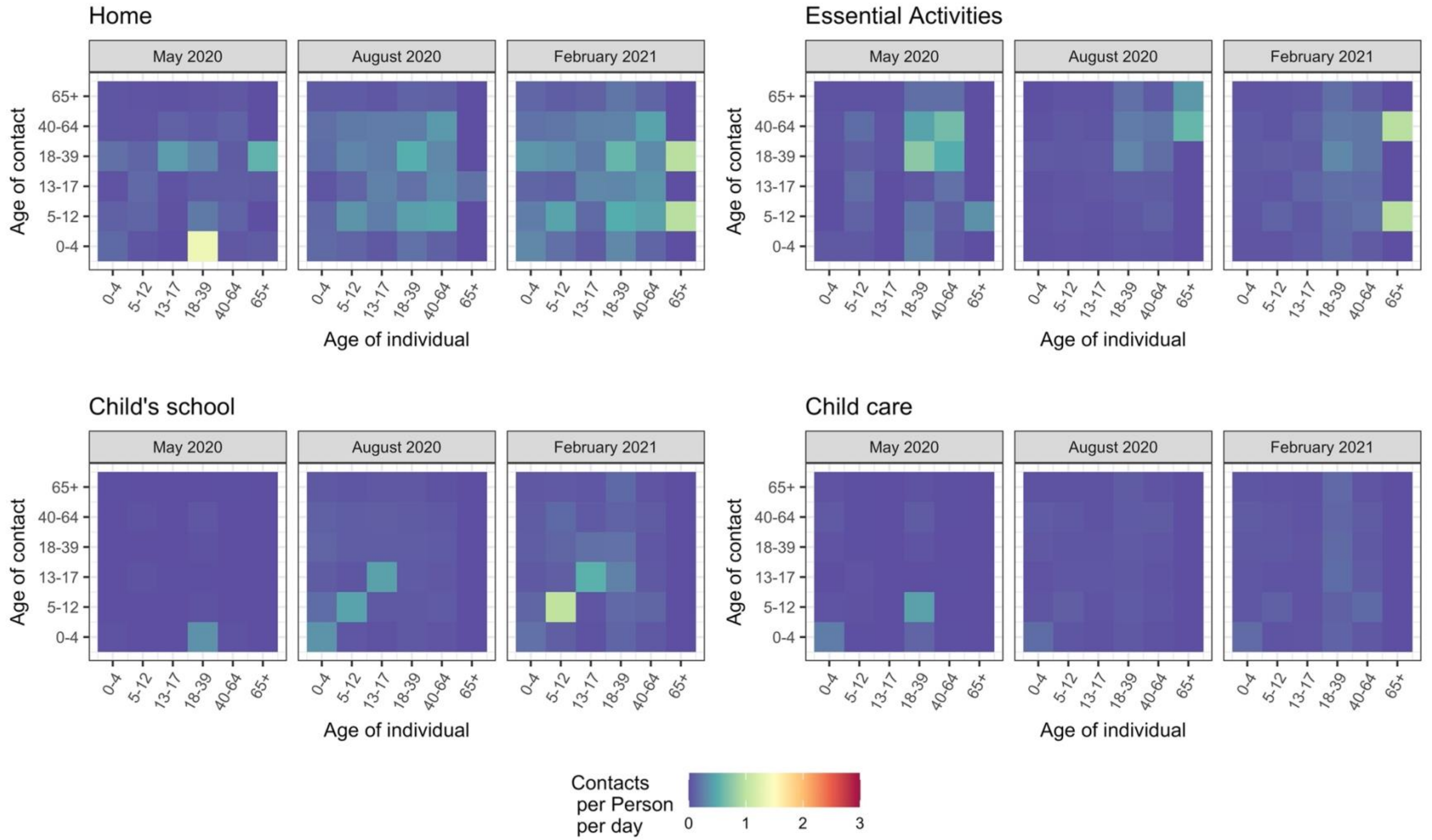

Figure S1. Age structured social contact matrices stratified by location and wave of data collection. May 2020 corresponds to wave one (May 4 – June 1, 2020), August 2020 corresponds to wave two (August 20 – October 1, 2020), and February 2021 corresponds to wave three (February 08 – April 07, 2021).

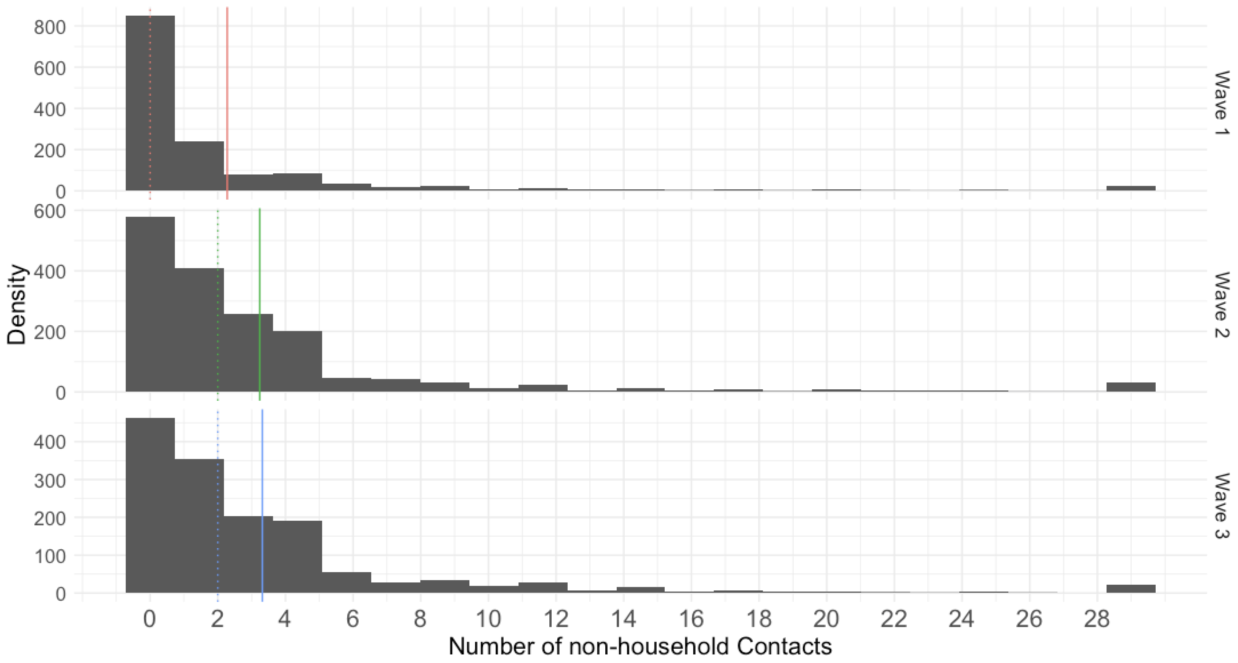

Figure S2. Histogram of total number of non-household contacts by study wave. The mean per study wave is indicated with the dashed line, and the median is indicated with the solid line.

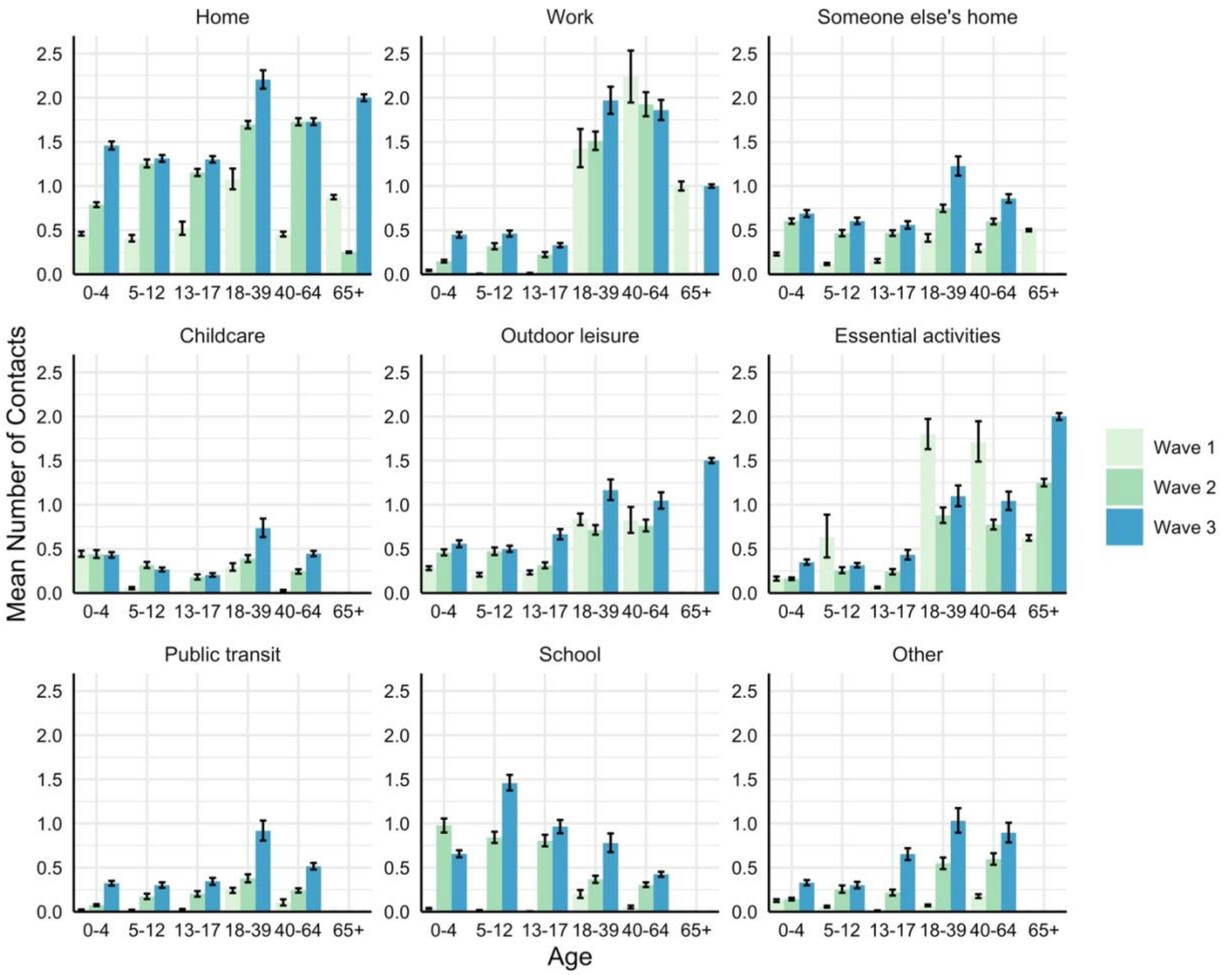

Figure S3. Average number of non-household contacts stratified by location of contact, age category, and study wave. Estimates were bootstrapped across 10,000 samples, and we present the 97.5% and 2.5% quantiles as 95% Confidence Intervals.

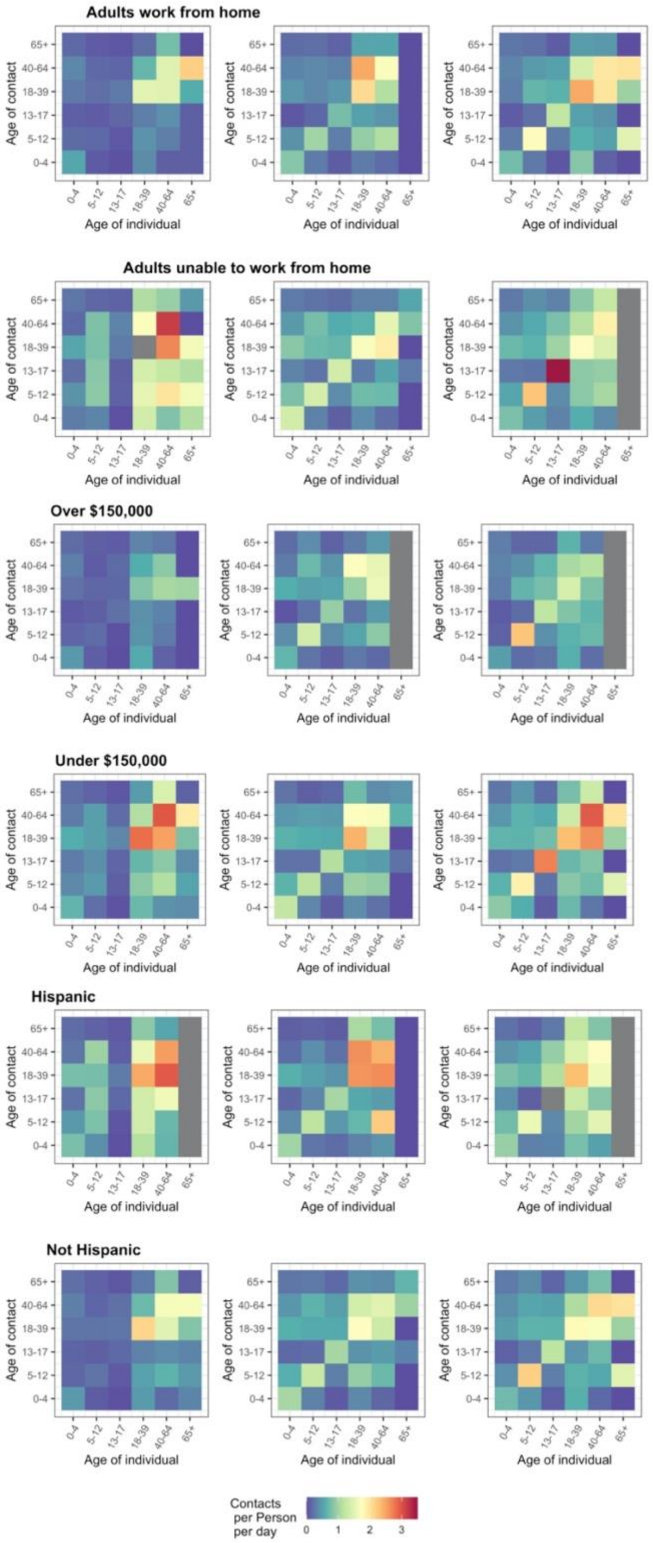

Figure S4. Age structured contact matrices stratified by household characteristic and BACK study wave. Estimates from wave one are presented in the first column, wave two in the middle, and wave three in the third column.

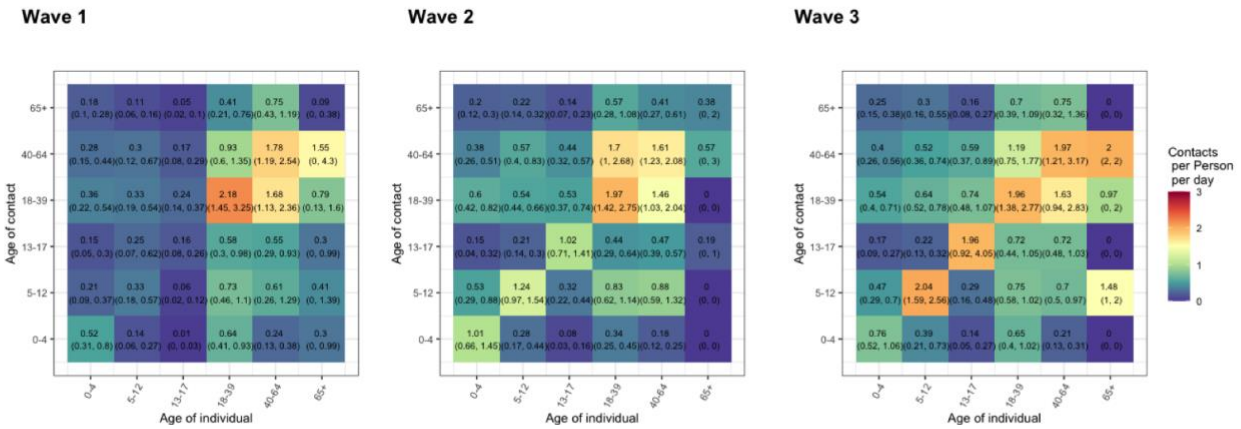

Figure S5. **Age-structured contact matrices populated with bootstrapped confidence intervals**. Estimates were calculated using the 2.5% and 97.5% quantile of 10,000 bootstrapped samples.

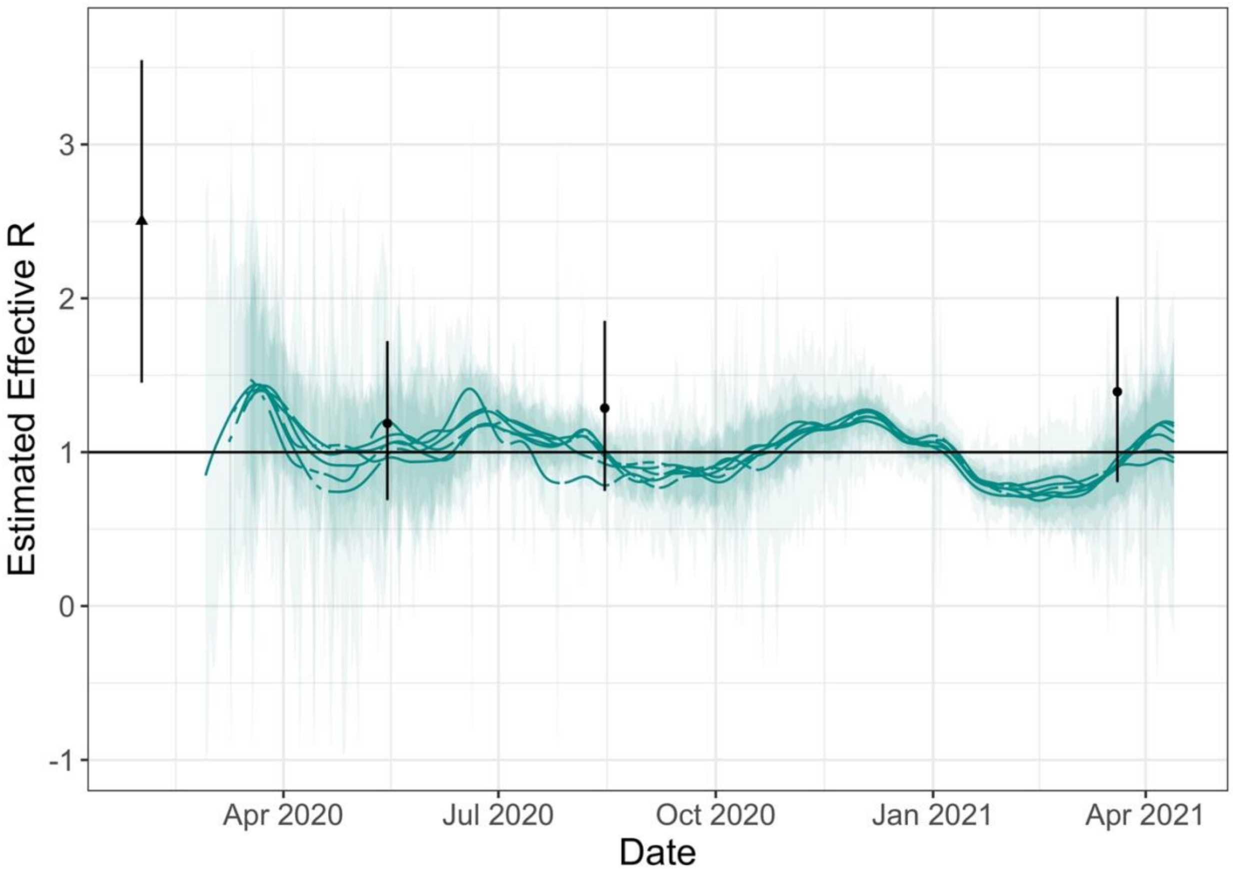

Figure S6. R0 estimates and 95% CIs from the BACK contact matrices for each wave of data collection. Estimates assume baseline contact patterns from the UK POLYMOD study and a baseline R_0 value drawn from a normal distribution with mean 2.5 and standard deviation 0.54 (triangle). Teal lines represent estimates of R_0 calculated from the incidence rates in 6 Bay Area counties, with shaded teal region representing the confidence intervals around these estimates.
